# Gut Microbiota Has a Widespread and Modifiable Effect on Host Gene Regulation

**DOI:** 10.1128/mSystems.00323-18

**Published:** 2019-09-03

**Authors:** Allison L. Richards, Amanda L. Muehlbauer, Adnan Alazizi, Michael B. Burns, Anthony Findley, Francesco Messina, Trevor J. Gould, Camilla Cascardo, Roger Pique-Regi, Ran Blekhman, Francesca Luca

**Affiliations:** a Center for Molecular Medicine and Genetics, Wayne State University, Detroit, Michigan, USA; b Department of Obstetrics and Gynecology, Wayne State University, Detroit, Michigan, USA; c Department of Genetics, Cell Biology and Development, University of Minnesota, Minneapolis, Minnesota, USA; d Department of Ecology, Evolution and Behavior, University of Minnesota, Minneapolis, Minnesota, USA; e Department of Biology, Loyola University Chicago, Chicago, Illinois, USA; University of Pennsylvania

**Keywords:** microbiome, gene expression, genomics

## Abstract

Variation in gut microbiome is associated with wellness and disease in humans, and yet the molecular mechanisms by which this variation affects the host are not well understood. A likely mechanism is that of changing gene regulation in interfacing host epithelial cells. Here, we treated colonic epithelial cells with live microbiota from five healthy individuals and quantified induced changes in transcriptional regulation and chromatin accessibility in host cells. We identified over 5,000 host genes that change expression, including 588 distinct associations between specific taxa and host genes. The taxa with the strongest influence on gene expression alter the response of genes associated with complex traits. Using ATAC-seq, we showed that a subset of these changes in gene expression are associated with changes in host chromatin accessibility and transcription factor binding induced by exposure to gut microbiota. We then created a manipulated microbial community with titrated doses of *Collinsella*, demonstrating that manipulation of the composition of the microbiome under both natural and controlled conditions leads to distinct and predictable gene expression profiles in host cells. Taken together, our results suggest that specific microbes play an important role in regulating expression of individual host genes involved in human complex traits. The ability to fine-tune the expression of host genes by manipulating the microbiome suggests future therapeutic routes.

**IMPORTANCE** The composition of the gut microbiome has been associated with various aspects of human health, but the mechanism of this interaction is still unclear. We utilized a cellular system to characterize the effect of the microbiome on human gene expression. We showed that some of these changes in expression may be mediated by changes in chromatin accessibility. Furthermore, we validate the role of a specific microbe and show that changes in its abundance can modify the host gene expression response. These results show an important role of gut microbiota in regulating host gene expression and suggest that manipulation of microbiome composition could be useful in future therapies.

## Abstract (precis)

The composition of the gut microbiome has been associated with various aspects of human health, but the mechanism of this interaction is still unclear. We utilized a cellular system to characterize the effect of the microbiome on human gene expression. We showed that some of these changes in expression may be mediated by changes in chromatin accessibility. Furthermore, we validate the role of a specific microbe and show that changes in its abundance can modify the host gene expression response. These results show an important role of gut microbiota in regulating host gene expression and suggest that manipulation of microbiome composition could be useful in future therapies.

## INTRODUCTION

The microbial community in the human digestive tract, the gut microbiota, is highly complex and also displays strong variation across individuals (1–3). Variability in gut microbiota composition is related to many factors, including medication, diet, and genetics (4–16). The gut microbiota has a variety of functions within the host, such as metabolism of certain compounds (17–20), and its composition is correlated with several diseases, such as Crohn’s disease and colorectal cancer (21–26). In mice, certain microbial communities can lead to changes in the host’s weight and overall health, suggesting that there is a reciprocal effect between the host and the gut microbiota (27). Recent work in mice has explored host gene expression and gene regulation in response to microbiome exposure (28–30). These studies suggested that the microbiome can induce both epigenetic changes and binding of specific transcription factors (28, 29, 31–33). In humans, work has been done to correlate microbiota composition, cellular response, and phenotype, thus illuminating the interplay between microbe and host. For example, studies have shown that expression and RNA splicing changes correlate with changes in the gut microbiota *in vivo* (34–37). However, in humans, it is challenging to perform large-scale studies and to account for environmental effects, such as host diet, health status, and medication use.

Recently, we described an *in vitro* approach based on human epithelial cells inoculated with live microbial communities (38) that is well suited to the study of the effects of the microbiome on human gene regulation. In short, we cultured human colonocytes under hypoxic conditions in order to recapitulate the gut environment. We then exposed the colonocytes to live gut microbiotas derived from human fecal samples under hypoxic conditions and measured the levels of response of host cells via transcriptome sequencing (RNA-seq). Using this technique, we identified differentially expressed (DE) genes that were enriched among genes associated with microbiome-related diseases, such as obesity (27, 39), and among genes that were differentially expressed in germ-free mice exposed to gut microbiota (28). The advantage of this system is that we can study human cellular response to gut microbiota in a highly scalable way.

Here we sought to use this *in vitro* system to determine the extent to which and the mechanism by which interindividual variations in microbiome composition drive differences in gene expression in the host cells. In order to assess the mechanism of cellular response to microbiotas, we considered chromatin accessibility via ATAC-seq (40), which utilizes the Tn*5* transposase for fragmentation and tagging of accessible DNA. Similar to DNase-seq, ATAC-seq can capture open chromatin regions, which are regions accessible to transcription factor (TF) binding. Changes in chromatin accessibility can modulate transcription factor binding and thereby influence gene expression (33, 41). The specific TFs binding in regions of open chromatin can be identified through the motif sequence model and footprinting analysis of ATAC-seq data. We also sought to determine if specific microbial taxa drive gene expression variation. These open issues are crucial for understanding the causal role of the microbiome in host physiology and for designing targeted therapies revolving around interventions in the gut microbiome.

## RESULTS

### Exposure to microbiota influences host gene expression.

**FIG 1:**
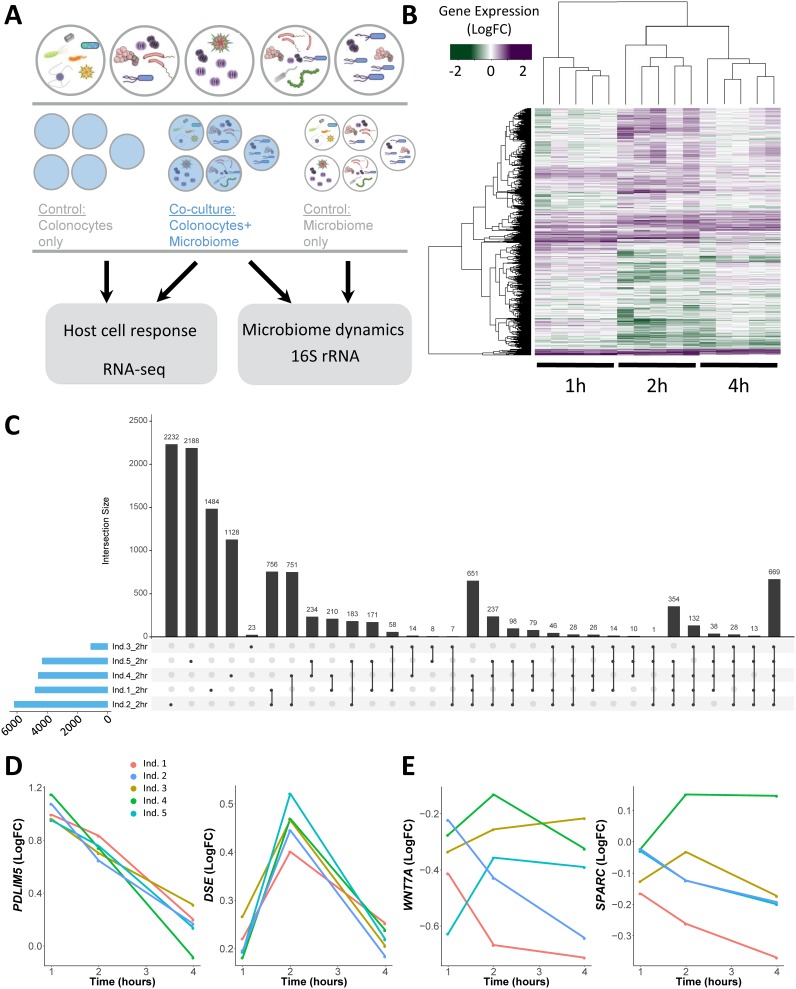
Gene expression changes in colonocytes treated with microbiota from five unrelated individuals. (A) Study design. Human colonocytes were inoculated separately with five microbiota samples from unrelated individuals. (B) Heat map of gene expression changes induced at each time point by the individual microbiota samples. Purple denotes an increase in gene expression (green shows a reduction) compared to the gene expression in the control (colonocytes cultured alone). Only genes that were differentially expressed in at least one sample are shown. LogFC, log fold changes. (C) Comparison of levels of transcripts differentially expressed at 2 h across the five treatments for the five individual microbiota samples (Ind.1 to Ind.5). The blue bars to the left show the total number of differentially expressed transcripts in the given set. The gray vertical bars show the number of transcripts that are in the set denoted below them. Sets with a single dark gray circle show the number of differentially expressed transcripts unique to that sample. (D) Examples of genes (*PDLIM5* [left panel] and *DSE* [right panel]) whose changes in expression were consistent across treatments with the five different microbiotas. Changes in expression (*y* axis) are shown as log_2_ fold change compared to control. (E) Examples of genes (*WNT7A* [left panel] and *SPARC* [right panel]) whose changes in expression were significantly different across treatments with the five microbiota samples.

To determine the impact of variation in the gut microbiota on host cells, we treated human colonic epithelial cells (HCoEpiC) with live gut microbiota extracted from 5 healthy, unrelated human individuals (Fig. 1A; see also Fig. S1A to D in the supplemental material). The composition of these samples is representative of that other healthy gut microbiome samples from the Human Microbiome Project (Fig. S1E) (1, 42). We then separately assessed changes in gene expression and microbial composition following 1, 2, and 4 h of exposure. The overall changes in gene expression among microbiome treatments and controls are clustered first by time point (by principal-component analysis and by hierarchical clustering as shown in Fig. 1B [see also Table S1 in the supplemental material]), where the strongest response (3,240 genes across any of the five microbiota samples) occurred at 2 h following exposure (see equation 3 in Text S1 in the supplemental material; Benjamini-Hochberg false-discovery rate [BH FDR] < 10%, |log_2_fold change [FC]| > 0.25). Among these, we identified 669 transcripts (188 genes) that were differentially expressed in all five treatments following 2 h of treatment (Fig. 1C; see also 1-h and 4-h comparisons in Fig. S1F and G). To identify genes whose expression levels changed consistently across the treatments at each time point, we removed the individual effect from the model and considered the 5 microbiota treatments as replicates. Two representative genes, *PDLIM5* and *DSE*, that were found to be differentially expressed at each time point are shown in Fig. 1D. Notably, the results from analysis of those genes show that we were able to identify various expression patterns through this model as long as the 5 microbiota treatments led to the same response at a given time point (Fig. S1H and I; see also Table S1). These 5,413 genes with shared expression changes were enriched for genes that function in protein translation, as well as those associated with the cell surface, such as in adherens junctions (BH FDR < 10^−6^) (Fig. S1J; see also equation 2 in Text S1), suggesting a biological function that may relate to the host cell’s interaction with the microbiota.

10.1128/mSystems.00323-18.1 TEXT S1 Supplemental methods and results: supplementary text for materials and methods. Download Text S1, PDF file, 0.2 MB. Copyright © 2019 Richards et al. 2019 Richards et al. This content is distributed under the terms of the Creative Commons Attribution 4.0 International license.

10.1128/mSystems.00323-18.2 FIG S1 Impact of microbiota exposure on host gene expression. (A) Relative abundances of the bacterial phyla in each of the five different microbial communities at 0 (uncultured), 1, 2, and 4 h with and without (+/-) coculturing with colonocytes. (B) Alpha diversity rarefaction plots for each of the microbial communities in this study. Error bars represent the standard deviations of the results from the multiple rarefaction trials. (C) Principal-coordinate analysis (PCoA; unweighted UniFrac) of the samples showed a clear separation by microbial community (left panel), while neither coculture conditions (center panel) nor time (right panel) had a dramatic effect on the community structures. (D) PCoA of samples using weighted UniFrac, colored by microbial community. (E) PCoA representing HMP samples and samples from the current study, with the HMP fecal samples indicated in red and all other HMP samples in gray. The five fecal samples used in the current study are indicated in yellow. The top panel shows PC1 (*x* axis) versus PC2 (*y* axis), while the bottom panel shows PC1 (*x* axis) versus PC3 (*y* axis). (F) Plots generated by the use of the UpSetR package to show comparisons of genes that were differentially expressed at 1 h following exposure to each of the five microbiota. (G) Comparison of differentially expressed genes across the five treatments at 4 h. (H) Plot generated by the use of the UpSetR package to show comparisons of genes across time points in the model where the five treatments were treated as replicates. The blue bars to the left indicated the total number of differentially expressed genes in the given set. The gray vertical bars show the number of genes that are in the set denoted below them. Sets with a single dark gray circle show the number of differentially expressed genes unique to that sample. (I) Heat map showing gene expression changes from the model in which the five microbiota treatments at a given time point were used as replicates. Purple denotes an increase in gene expression (green shows a reduction) compared to the gene expression in colonocytes cultured alone. Only genes that were differentially expressed in at least one time point are indicated here. (J) Plot showing gene ontology enrichment for genes that were differentially expressed at any time point in the model in which each treatment was considered to represent a replicate (and in which the treatments led to similar expression changes). The top 10 enrichments are shown in this plot, excluding categories where the expected number of genes was less than 10 or greater than 500. The size of the point corresponds to the –log_10_ of the adjusted *P* value. Download FIG S1, PDF file, 1.1 MB. Copyright © 2019 Richards et al. 2019 Richards et al. This content is distributed under the terms of the Creative Commons Attribution 4.0 International license.

10.1128/mSystems.00323-18.5 TABLE S1 Differentially expressed genes with various models. The table lists all transcripts that were differentially expressed in each of 5 models. (A) Model considering transcripts that were DE at each time point and with each treatment. (B) Model considering transcripts that were DE at each time point (with the 5 microbiota treatments considered replicates). (C) Model considering transcripts that were DE across treatments as determined using the likelihood ratio test. (D) Model considering transcripts that were DE relative to the mean-centered baseline abundance of the denoted taxon. (E) Model considering transcripts that were DE following coculture of colonocytes with the microbiome plus various concentrations of Collinsella aerofaciens. Genes were considered differentially expressed if at least one transcript was differentially expressed. Download Table S1, TXT file, 16.8 MB. Copyright © 2019 Richards et al. 2019 Richards et al. This content is distributed under the terms of the Creative Commons Attribution 4.0 International license.

Each microbiota sample was derived from a different individual with a unique diet and genetic makeup. Therefore, we expect that the microbial composition and diversity of all samples differ. Examining the uncultured microbiomes, we found variability in their microbial composition and diversity (Simpson’s index values ranged between 0.94 and 0.98). In contrast, we found that the microbial communities in the cultured microbiomes did not change dramatically over time and that the microbiomes maintained their individual-specific composition characteristics during culture (Fig. S1A to C). Next, we considered how the human colonocytes influence microbial composition, and we found that most taxa were unaffected by the presence of human cells, while 13 taxa (of 112 tested) showed various levels of abundance dependent on the presence of host cells (likelihood ratio test; BH FDR < 10%) (examples are shown in Fig. S2A and B, table shown in Fig. S2C). In order to determine how the compositions of the microbiota samples influenced host gene expression differently, we utilized a likelihood ratio test to compare models that included or excluded the individual microbiota effect. This test is able to incorporate gene expression changes over time and to compare the trajectories of expression in response to the 5 different microbiota treatments. In this way, we identified 409 genes (1,484 transcripts) (likelihood ratio test; BH FDR < 10%; see Table S1) with expression patterns that significantly differed in response to the five microbiota samples. Two examples of representative genes with differing gene expression patterns are *WNT7A* and *SPARC* (Fig. 1E). These examples demonstrate that no two microbiota samples induce the same response in the genes identified by the likelihood ratio test and further showed that the genes had different responses to the same treatment. These data demonstrate that the host and the microbiota influence each other and that interindividual variation in the microbiome can lead to different gene expression responses in interacting host cells.

**FIG 2:**
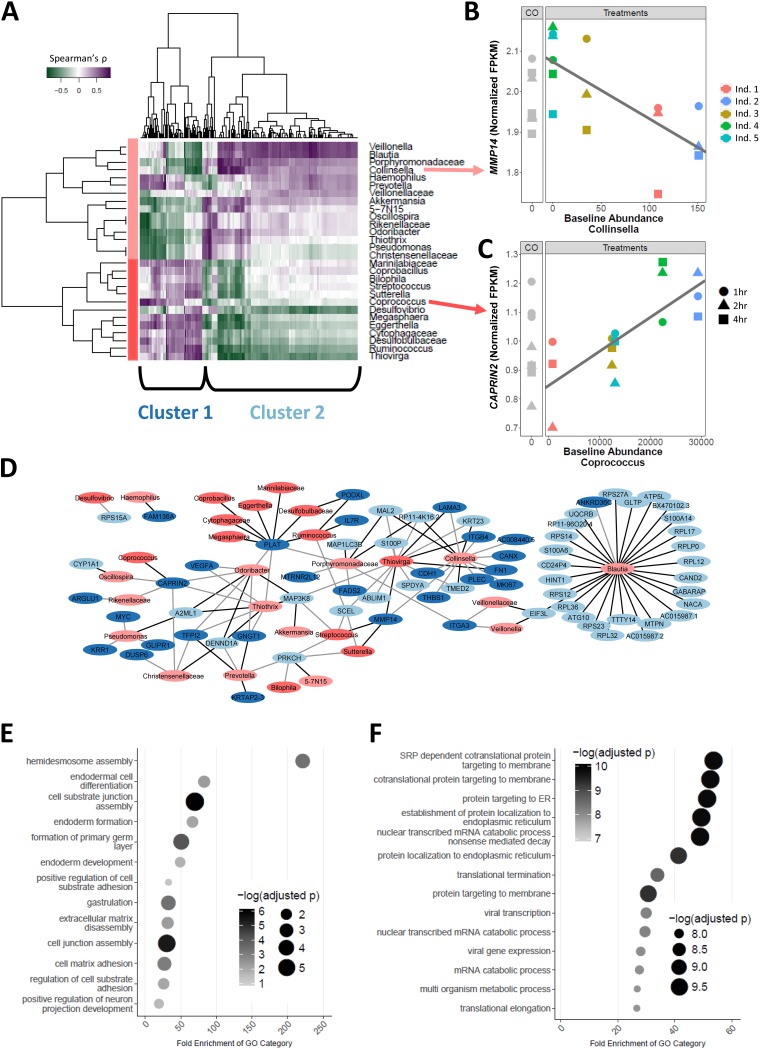
Abundance of microbiome taxa is associated with specific host gene expression changes. (A) Heat map of microbiota taxa and colonocyte gene expression correlation (Spearman’s ρ). Rows correspond to 28 microbiota taxa (which include all OTUs within the taxa if collapsed to genus level), and columns correspond to 219 transcripts (70 genes) that had at least one significant association (likelihood ratio test [LRT]). Taxa and transcripts are each clustered via hierarchical clustering, showing two major groups indicated by a different shade of red (taxa)/blue (transcripts). (B and C) Examples (*MMP14* [B] and *CAPRIN2* [C]) of significant association (BH FDR = 7% for both genes) between host gene expression (fragments per kilobase per million [FPKM] quantile normalized) and baseline abundance of specific taxa. (CO), control samples. (D) Network of associations between taxa and genes from the heat map. Nodes in blue denote genes, while nodes in red denote microbial taxa. Color shading indicates clusters of genes or taxa as defined in the heat map. Black edges indicate a positive correlation, while light gray indicates a negative correlation. (E and F) Gene ontology enrichment for cluster 1 (E) and cluster 2 (F). ER, endoplasmic reticulum; SRP, signal recognition particle.

10.1128/mSystems.00323-18.3 FIG S2 Changes to the microbiome and concordance of taxon model and *Collinsella* spike-in. (A and B) Genera (*Faecalibacterium* [A] and *Bacteroides* [B]) whose changes in abundance following coculturing were inconsistent in a manner dependent on the microbiota sample in which they were found. Each color denotes samples exposed to a particular microbiota sample as follows: red, Ind1; blue, Ind2; yellow, Ind3; green, Ind4; teal, Ind5. (C) Table denoting 13 taxa whose abundance was affected by culturing with colonocytes as determined by the likelihood ratio test in comparisons between models with and without a treatment effect (BH FDR < 10%). (D and E) Correlations within the microbial taxon clusters shown in Fig. 2A. (D) Microbes positively associated with genes in cluster 2. (E) Microbes associated with genes in cluster 1. Red edges indicate a positive correlation. Blue edges indicate a negative correlation. Edge thickness indicates the magnitude of the correlation. Correlations and corresponding significance data from comparisons between pairs of microbial taxa were generated using SparCC, which estimates linear Pearson correlations, with 100 bootstrap iterations (72). Correlations were filtered by the use of a BH FDR of <5% and a correlation magnitude of >0.5. (F) QQ plot of *P* values from the model of the five-microbiota experiment corresponding to abundances of baseline *Collinsella* (gray). The red points indicate the same values as those indicated in gray but that were grouped in subsets to only include the 1,570 differentially expressed genes (DEG) in the validation spike-in experiment performed with Collinsella aerofaciens. Download FIG S2, PDF file, 0.2 MB. Copyright © 2019 Richards et al. 2019 Richards et al. This content is distributed under the terms of the Creative Commons Attribution 4.0 International license.

### Specific microbes influence unique host genes.

We hypothesized that the differences in gene expression responses to each microbiome could be attributed to specific microbiota features, such as the abundance of specific taxa. For this reason, we used DESeq2 to study the association between host gene expression (147,555 transcripts) and the abundance of microbial taxa (62 taxa that passed filtering criteria; see Materials and Methods and Text S1) at the time of treatment. Across all possible associations (9,125,927 tests), we identified 588 transcript-by-taxon pairs with a significant association (see equation 5 in Text S1; BH FDR = 10%) (see also Table S1), corresponding to 121 host genes with changes in expression associated with the abundance of 46 taxa. Among those taxa, 35 were associated with the expression of two or more host genes (see equation 5 in Text S1; BH FDR = 10%), suggesting that a single microbe may affect the regulation of many genes and the presence of a *trans* regulatory mechanism by which microbes may influence host organism traits.

Of the 121 host genes whose expression was found to be associated with abundance of microbial taxa, 70 genes (219 transcripts) were also found to be differentially expressed in comparisons of microbiota treatment to control and formed two clusters with distinct functions (Fig. 2A and D; examples are shown in Fig. 2B and C). Genes in the first cluster were found to be positively correlated with genera *Ruminococcus*, *Coprococcus*, and *Streptococcus* and to have functions in cell junction assembly (GeneTrail enrichment analysis BH FDR < 10^−5^) (Fig. 2E), while those in the second cluster were found to be positively correlated with microbial genera that included *Odoribacter*, *Blautia*, and *Collinsella* and to function in protein targeting of the endoplasmic reticulum (GeneTrail enrichment analysis BH FDR < 10^−7^) (Fig. 2F). Our analyses of the abundances of the microbes in the clusters showed them to be highly correlated, further suggesting that the microbial community as a whole may act on host gene expression (Fig. S2D and E).

**FIG 3:**
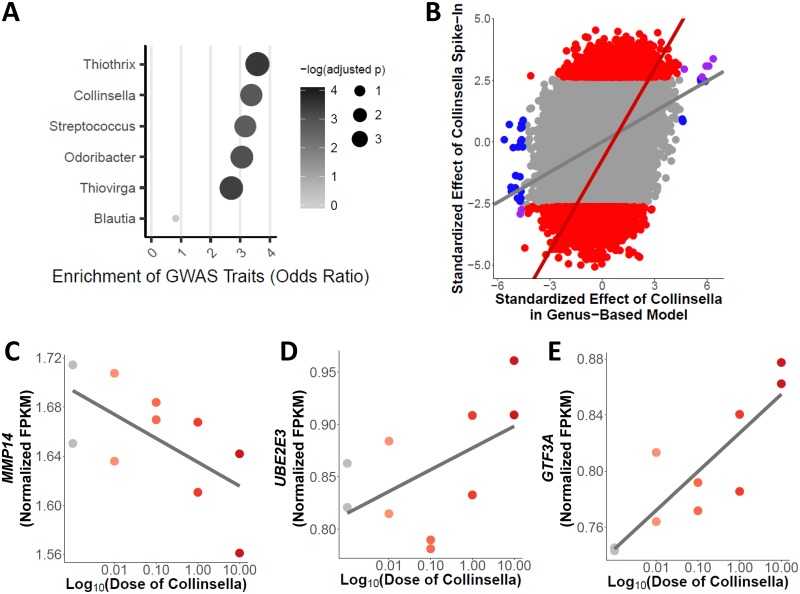
Manipulation of the microbial community induces predictable gene expression changes in the host. (A) Genes associated with particular microbiota (BH FDR < 20%) were enriched for complex traits. Microbiota were chosen if they had at least 30 genes associated via the genus-based model for gene expression. The shade of the point indicates the −log(adjusted *P* value) from the enrichment test, while the size of the point and the *x*-axis values give the fold enrichment. (B) Scatterplot of the effect of *Collinsella* abundance from the five microbiome samples (*x* axis) and effect of Collinsella aerofaciens from the spike-in validation experiment (*y* axis). Plotted are log_2_ fold changes normalized by the standard error. Blue points denote transcripts that were DE only in the genus-based model that included the abundance of *Collinsella*. The red points and line highlight the transcripts that were DE only in the spike-in experiment. Purple points denote transcripts that were DE in both the genus-based model and the spike-in experiment. Gray points denote transcripts that were not differentially expressed in either the gene-based model with abundance of *Collinsella* or in the spike-in experiment. There was a correlation across all points (linear regression *P* value < 10^−20^, Spearman’s ρ = 0.29) and across transcripts differentially expressed only in the spike-in experiment (linear regression *P* value < 10^−20^, Spearman’s ρ = 0.46). (C to E) Examples (*MMP14* [C], *UBE2E3* [D], and *GTF3A* [E]) of significant association (DESeq2 BH FDR = 9%, 6%, and 7%, respectively) between host gene expression (FPKM quantile normalized) and abundance of Collinsella aerofaciens from spike-in validation experiment (linear regression *P* value = 0.05, 0.02, and 0.004 with Pearson’s *r* = –0.64, 0.73, and 0.81, respectively).

We then focused on microbial taxa associated with changes in expression for a large number of genes, since these microbes are more likely to impact host traits. To test this hypothesis, we focused on the six microbial genera that were associated with the largest number of host genes (at least 30 host genes at *P* values of <3.5 × 10^−5^ [see equation 5 in Text S1]): *Odoribacter*, *Streptococcus*, *Blautia*, *Thiovirga*, *Thiothrix*, and *Collinsella*. Indeed, all of those taxa, except for *Blautia*, showed expression changes in genes that were between 2.7-fold and 3.6-fold enriched (compared to a background of expressed genes) for those associated with complex traits from the genome-wide association study (GWAS) catalog (Fisher’s exact test *P* value < 0.005) (Fig. 3A; see also Table S2) (43). Moreover, we identified 21 genes that were associated with traits already linked to the gut microbiome, including colorectal cancer (21, 26), obesity (27, 39), and inflammatory bowel disorder (IBD) (44–46). Genes associated with the genus *Collinsella* were found to be 3.4-fold enriched for those associated with a trait in GWAS (15 of 30 genes; Fisher’s exact test *P* value = 0.001) (Fig. 3A). Previous studies revealed that the abundance of *Collinsella* is correlated with several diseases, including colorectal cancer (47), Type 2 Diabetes (48), and irritable bowel syndrome (49). Interestingly, we identified a gene, *GLTP*, that is involved in glycolipid transfer and that has been associated with metabolic syndrome (50). Based on the transcriptome-wide association study (TWAS) analysis reported in Metaxcan (51–53) (https://imlab.shinyapps.io/metaxcan_a_1/), higher expression of GLTP is associated with decreased levels of high-density lipoprotein (HDL) (*P* value = 3.1 × 10^−7^), which is a factor contributing to metabolic syndrome. In our data, the abundance of the genus *Collinsella* was found to be positively associated with GLTP expression (see equation 5 in Text S1; BH FDR = 12.6%). Additionally, GLTP was shown expressed in the colon according to the GTEx data (average number of transcripts per million [TPM] = 47 in transverse colon and 39 in sigmoid colon). These data suggest an association between *GLTP* and metabolic syndrome that may be modulated by exposure to *Collinsella*. These data also suggest that specific microorganisms, and not simply general exposure to the entire gut microbiota, can lead to changes in the expression levels of many genes. Furthermore, these results support the hypothesis that variations in the abundances of members of the microbiota may influence complex traits.

10.1128/mSystems.00323-18.6 TABLE S2 DE genes from taxon model found associated with GWAS traits. The table shows the gene symbol and Ensembl identifier (ID) for genes identified and differentially expressed in the genus-based model. The third column shows the genera with which gene expression was associated, and the fourth column delineates the traits and the papers where the gene was reported to be associated with a GWAS trait. Download Table S2, TXT file, 0.01 MB. Copyright © 2019 Richards et al. 2019 Richards et al. This content is distributed under the terms of the Creative Commons Attribution 4.0 International license.

### Validation of changes in host gene expression due to specific microbiota.

To validate and further demonstrate the effect of specific microbes on host gene expression, we treated colonocytes with a microbiota sample without any detectable Collinsella aerofaciens and supplemented it with titrated abundances (0.01%, 0.1%, 1%, and 10%) of this bacterium relative to the whole-microbiota sample. We used RNA-seq to study the resulting changes in gene expression and identified 1,570 genes whose expression levels had changed (see equation 6 in Text S1; BH FDR = 10%) (Table S1; see also Fig. S2F) in a manner dependent on the abundance of Collinsella aerofaciens. Examining changes in gene expression associated with *Collinsella* abundance in the five microbiota treatments, we found that the effects of *Collinsella* in the two experiments were correlated (Fig. 3B). We validate 19 of the 29 genes originally identified (Fisher’s exact test *P* value = 0.0002, odds ratio [OR] = 4.1) (see equation 5 in Text S1; BH FDR = 20%), including *GLTP* and *MMP14* (see equation 5 in Text S1; BH FDR = 7% in Fig. 2B) (see also equation 6 in Text S1; BH FDR = 9% in Fig. 3C), demonstrating that the presence of *Collinsella* is sufficient for the occurrence of changes in the expression of these genes. Interestingly, we also found a correlation between the effect of *Collinsella* in the spike-in experiment and the effect of several other microbes from our genus-based model on host gene expression. Specifically, we found positive correlations of effects on gene expression between the *Collinsella* spike-in experiment and other microbes in the cluster that contained *Collinsella* (Fig. 2A; Spearman’s ρ = 0.25 to 0.4) and negative correlations for microbes in the cluster that did not contain *Collinsella* (Spearman’s ρ = –0.27 to –0.32). These results underline the importance of considering the entire microbial community rather than individual microbial species in studying host-microbiome interactions. That the expression levels of a large number of genes changed in this experiment could have been due to several factors, including the increase in the power of the analysis that resulted from the use of a larger number of samples. These 1,570 genes were found to be enriched for those associated with complex traits in the GWAS data compared to a background of all expressed genes (Fisher’s exact test *P* value = 10^−10^, OR = 1.5) and to be specifically enriched for genes associated with HDL cholesterol (Fisher’s exact test, Bonferroni-corrected *P* value = 0.018, OR = 2.75). Other DE genes were found to be associated with additional traits relevant to the microbiome, such as body mass index (e.g., see *GTF3A* in Fig. 3E) and obesity and celiac disease (e.g., see *UBE2E3* in Fig. 3D). The results of this spike-in experiment showed that host gene expression can be modulated by changing the abundance of a single bacterial species within the microbiome.

### Changes in chromatin accessibility and transcription factor binding following microbiota exposure.

**FIG 4:**
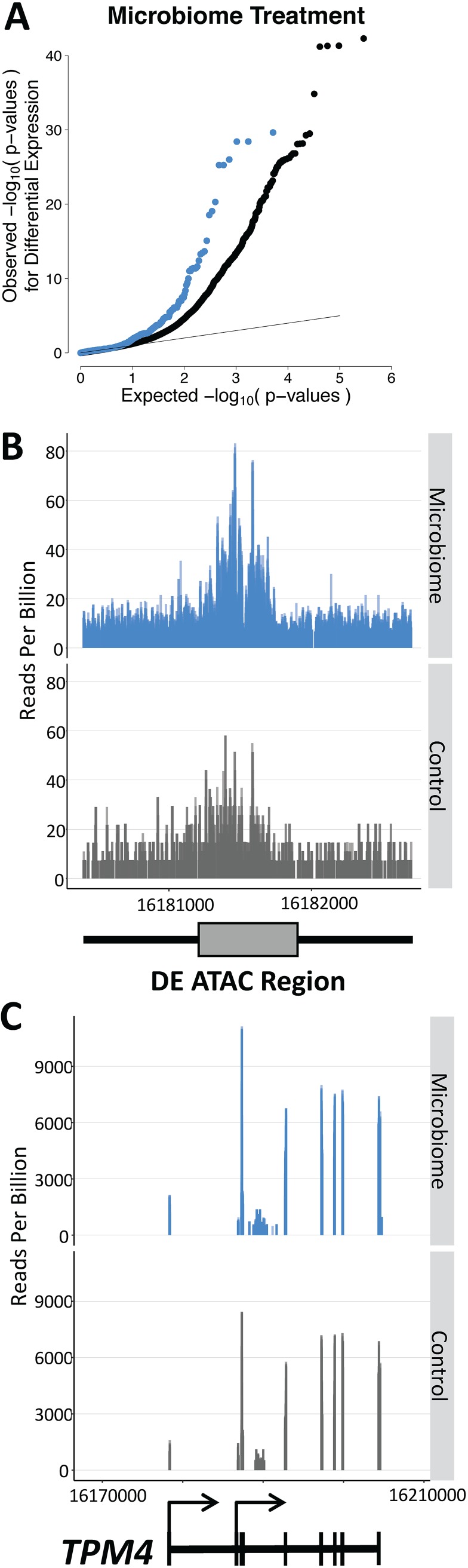
The microbiome induces changes in gene expression through changes in chromatin accessibility. A QQ (quantile-quantile) plot of *P* values from the analysis of consistent differential levels of gene expression in colonocytes treated with five microbiota samples for 4 h. The blue points indicate the *P* values for transcripts that were within 50 kb of a differentially accessible region identified through ATAC-seq after 2 h of treatment. The gray points indicate transcripts that were not within 50 kb of a differentially accessible region. (B) ATAC-seq profile centered on the 300-bp window (with 1,000 bp on either side) that was differentially accessible following 2 h of treatment with the microbiome (log_2_ fold change [FC] = 0.47, BH FDR = 12%). This region was found 4,795 bp distant from the differentially expressed *TPM4* gene. (C) RNA-seq profile of *TPM4* (ENST00000586833) (and of 20,000 bp in either direction), which was differentially expressed following 4 h of treatment with the microbiome (log_2_ FC = 0.29, BH FDR = 1.9%). The gene model is shown at the bottom. Panels B and C show the summation of reads at each position across the five biological replicates and the two technical replicates.

In order to investigate the regulatory mechanism whereby the microbiome induces changes in host gene expression, we performed ATAC-seq in colonocytes inoculated for 2 h with each of the five microbiota samples and were able to identify ATAC-seq peaks at the transcription start site (TSS) of expressed genes (Fig. S3E). We then used DESeq2 to characterize regions with differential levels of chromatin accessibility. We identified only a limited number of regions that were differentially accessible across the five microbiome treatments with two technical replicates (*n* = 234) (Fig. S3A). Nevertheless, we found enrichment of differentially accessible regions at 2 h within 50 kb of genes differentially expressed at 4 h (Fig. 4A; see also Table S3) (Fisher’s exact test *P* value = 3.96 × 10^−4^, OR = 2.13). We note that this enrichment consisted of 24 differentially accessible chromatin regions within 50 kb of 32 differentially expressed genes and so still represented a minority of the differentially expressed genes. Interestingly, we did not find the same enrichment when we examined genes differentially expressed at 1 and 2 h following exposure to gut microbiota (Fisher’s exact test *P* value > 0.15), suggesting that the changes in chromatin accessibility that we identified occurred first (at 2 h) and then led to subsequent changes in gene expression by 4 h postinoculation.

10.1128/mSystems.00323-18.4 FIG S3 Effect of microbiome exposure on host chromatin accessibility. (A) We identified changes in chromatin accessibility between microbiome-exposed samples and control samples for each replicate and show that these changes are highly correlated (*r*^2^ = 0.835, *P* value < 2.2 × 10^−16^). Points shown here denote positions that we identified as being differentially accessible when the replicates are used together in the same model. Gray points denote regions that, considered alone, were not considered differentially accessible in either replicate. Blue and red points denote regions that were differentially accessible in one replicate (BH FDR < 20%), and purple points denote regions that, even for replicates considered separately, were considered differentially accessible in both replicates. (B) Each point denotes a differentially accessible ATAC region and a differentially expressed gene within 50 kb of each other (total, 36 pairs). The transcript that was most significantly differentially expressed after 4 h was used to represent each gene. The plot shows the correlation of changes in chromatin accessibility via ATAC-seq and changes in gene expression of a gene via RNA-seq (*r*^2^ = 0.647, *P* value = 3.361 × 10^−9^). *TPM4* is denoted in blue. (C) ATAC-seq profile centered on the 300-bp window (with 1,000 bp on either side) that was differentially accessible following 2 h of treatment with the microbiome (BH FDR = 12%). This region was found 4,795 bp from the differentially expressed *TPM4* gene. (D) RNA-seq profile of *TPM4* (ENST00000586833) (and of 20,000 bp in either direction), which was differentially expressed following 4 h of treatment with the microbiome (BH FDR = 1.9%). The gene model is shown below. The tracks shown in panels C and D are equivalent to the averaged tracks in Fig. 4B and C, except that here they are separated by sample. Each color denotes a different treatment as follows: control colonocytes are shown in gray and colonocytes that received treatment with microbiota derived from each of the five individuals (“Ind”) in a shade of blue. Technical replicates (“rep”) are coded in the same color shade. The same colors (indicating treatment) are used in panels C and D. (E) For each of the five samples and the control, we show the average number of ATAC-seq reads per million (RPM) at each base position within 1 kb upstream and downstream of the transcriptional start site for all genes expressed at 4 h. This plot was generated with metagene (73). (F) Gel with five wells: (i) 100-bp ladder, (ii) Collinsella aerofaciens DNA and (iii) Odoribacter splanchnicus DNA with primers specific to 16S region of Collinsella aerofaciens (590 bp), and (iv) Collinsella aerofaciens DNA and (v) Odoribacter splanchnicus DNA with primers appropriate for amplification of the 16S region in any microbial sample. Download FIG S3, PDF file, 0.4 MB. Copyright © 2019 Richards et al. 2019 Richards et al. This content is distributed under the terms of the Creative Commons Attribution 4.0 International license.

10.1128/mSystems.00323-18.7 TABLE S3 Differentially accessible regions within 50 kb of differentially expressed genes. The table denotes all transcript-by-ATAC region pairs for which both region and transcript were differentially expressed following treatment with the microbiome using the model where we considered the 5 microbiota treatments to be replicates. Download Table S3, TXT file, 0.03 MB. Copyright © 2019 Richards et al. 2019 Richards et al. This content is distributed under the terms of the Creative Commons Attribution 4.0 International license.

Notably, the directions of the changes in chromatin accessibility and in gene expression were concordant, which indicates that opening of the chromatin resulted in upregulation of gene expression, as expected (Fig. S3B). One interesting example is the *TPM4* gene, which is a gene that binds actin filaments and whose role in the regulation of the cytoskeleton may impact the host cell response to the microbiome. *TPM4* is regulated by the microbiome through changes in chromatin accessibility and showed increased expression at 4 h following treatment with the five microbiota samples (Fig. 4B and C; see also Fig. S3C and D). We identified the chromatin change at a region located at 4,800 bp upstream of the transcription start site of *TPM4*.

To identify transcription factors that may mediate the response to the microbiome, we performed footprinting analysis of the ATAC-seq data using CENTIPEDE (54). We identified 397 factors that were active under the treatment and control conditions, for a total of 36,565,954 and 28,922,773 footprints, respectively. We identified 40 motifs with footprints in differentially accessible regions, including factors that bind the ELF5, SOX2, and HMGA2 motifs (Table S4). Interestingly, using a stratified FDR approach to identify differential levels of chromatin accessibility for regions that contained footprints for active factors, we identified a larger number of differentially accessible regions (209 regions; FDR = 10%; Table S5).

10.1128/mSystems.00323-18.8 TABLE S4 Active motifs under treatment versus control conditions. We identified 40 motifs with footprints in differentially accessible regions, including factors that bind the ELF5, SOX2, and HMGA2 motifs. Motif enrichment or depletion in differentially accessible regions (DARs) was calculated using Fisher’s exact test for each motif. Each row of the table represents a significantly enriched or depleted motif. Column heading designations are defined as follows: Motif, name of motif; Odds_Ratio, odds ratio corresponding to enrichment or depletion; Lower, lower bound of 95% confidence interval; Upper, upper bound of 95% confidence interval; Enrichment.Pvalue, *P* value of odds ratio; TFpos_DARpos, number of DARs containing footprint; TFpos_DARneg, number of non-differentially accessible regions containing footprint; TFneg_DARpos, number of DARs not containing footprint; TFneg_DARneg, number of non-differentially accessible regions not containing footprint; Treatment_foots, total number of footprints under the indicated treatment conditions; Control_foots, total number of footprints under the control conditions. Download Table S4, TXT file, 0.01 MB. Copyright © 2019 Richards et al. 2019 Richards et al. This content is distributed under the terms of the Creative Commons Attribution 4.0 International license.

10.1128/mSystems.00323-18.9 TABLE S5 Differentially accessible regions by stratified FDR. The table lists 209 differentially accessible regions (DARs) from a stratified FDR test performed with ATAC-seq (BH FDR < 0.1). Each row of the table represents a DAR. Column heading designations are defined as follows: Chr, chromosome; Start, beginning of DAR; End, end of DAR; baseMean, mean of normalized ATAC-seq read counts across all samples, normalized for sequencing depth; log2FC, log_2_-transformed fold change; lfcSE, standard error of log fold change; stat, Wald statistic (log2 FC/lfcSE); p.val, *P* value of region being differentially accessible; DESeq_padj, nonstratified FDR adjustment; motifs, list of all motifs with footprint in region. Download Table S5, TXT file, 0.03 MB. Copyright © 2019 Richards et al. 2019 Richards et al. This content is distributed under the terms of the Creative Commons Attribution 4.0 International license.

We next investigated how specific transcription factors may be involved in modulating gene expression responses to the microbiome that may not require changes in chromatin accessibility. In this case, we conducted an enrichment analysis using a logistic model to predict which genes would be found to be differentially expressed, using the footprint counts for each of the 397 active factors within 50 kb of the gene transcription start site. At a FDR of 10%, we detected 110 factors that had footprints enriched in genes that were more likely to be differentially expressed after treatment, including motifs such as AP1, RELB, NκFB, STAT1, STAT3, and HNF4A (Table S6). Many of these factors are involved in immune response pathways, inflammation pathways, and chemokine signaling and may not require chromatin remodeling to activate their target genes to elucidate a response after microbiome treatment.

10.1128/mSystems.00323-18.10 TABLE S6 Transcription factor footprints enriched in differentially expressed genes. Using a logistic model, we identified motifs for transcription factors with footprints overrepresented or underrepresented on genes that were differentially expressed after microbiome treatment. Column heading designations are defined as follows: Motif, name of motif; Factor.Name, name of the transcription factor; estimate, estimated size of effect on the number of footprints; std.err, standard error; z_value, Z score; Pval, *P* value; BH.padj, multiple-test-adjusted *P* value determined using the Benjamini-Hochberg procedure. Download Table S6, TXT file, 0.04 MB. Copyright © 2019 Richards et al. 2019 Richards et al. This content is distributed under the terms of the Creative Commons Attribution 4.0 International license.

## DISCUSSION

The gut microbiota has recently been reported to be associated with several different diseases and disorders (21, 26, 27, 39, 44–46). However, the mechanism of action is not well understood, and we know little about how microbiome risk factors interact with the host genes that have been linked to the same disorders. Here, we investigated how interindividual variations in microbiome composition affect host gene regulation. Previous work in mice has shown that transplantation with microbiomes derived from mice with different phenotypes, e.g., obese versus lean, can lead to differences in organismal phenotypes (39). In contrast, our work focuses on variable responses associated with microbiomes derived from healthy humans. We note that the *in vitro* system has several limitations: for example, the presence of a low level (5%) of oxygen may be deleterious to obligate anaerobes. Moreover, the combination of the inclusion of a single cell type and the short duration of treatment (up to 4 h) does not recapitulate *in vivo* systems where microbes and host cells of different types interact for longer time periods. Nevertheless, this is a well-controlled system that is not susceptible to many of the biases that are found in other model systems and that allows characterization of the direct effect of microbial communities and isolates on gene regulation in host cells. We illustrate that while there were thousands of genes that showed consistent responses induced by the five microbiome treatments, there were several hundred that responded differently to each microbiome. Furthermore, some of the differences in response can be understood as having resulted from the variations in the microbiome compositions of the five samples. Though we focus on healthy microbiomes, our results highlight the interactions of particular microbes and host genes that may play roles in various host traits. For example, we identified hundreds of genes that responded to particular microbes in a predictable way, such as the *GLTP* gene, whose response was correlated with the presence of *Collinsella*. Our results suggest that *Collinsella* may impact complex traits through regulation of host gene expression. While we closely examined the effect of the presence of *Collinsella* on host gene expression, there were many other relevant microbes that may also play a role in host gene regulation. For example, the abundance of *Prevotella*, which has been associated with colorectal carcinoma (55) and ileal Crohn’s disease (56), induces changes in the expression of 6 genes (BH FDR = 10%). Further studies performed with a wider panel of microbiome samples will allow increased power to detect more genes that are differentially expressed across microbiome treatments.

One consideration in our analysis of the effect of *Collinsella* on host gene expression is that our treatments consisted of healthy microbiota samples spiked with *Collinsella*. This was done in order to better replicate natural variation in gut microbiota composition and microbial interaction in the human gut. Microbes are constantly in competition for particular niches in the gut, and so we do not expect that increases in *Collinsella* levels in an organism would occur alone but would instead likely affect the levels of other microbes. However, future work using treatments of *Collinsella* alone may identify genes whose expression levels change as direct consequences of *Collinsella* abundance rather than as a result of overall changes in microbial environment induced by manipulation of *Collinsella* abundance.

Our study of chromatin accessibility changes after 2 h of microbiome exposure in 12 samples identified a limited number of differentially accessible regions. These results are similar to those reported from previous work in conventionalized mice that did not show broad changes in chromatin accessibility compared to germ-free mice and could have been a consequence of limited statistical power, incorrect time point for detection, or short duration of the treatment (28, 29). In our study, the regions that were detected as significantly differentially accessible were enriched near genes that changed their expression following treatment with a consistent direction of effect; and yet the changes in chromatin accessibility were small and accounted for only a limited number of genes that were differentially expressed after treatment. Many of the changes in gene expression could still be mediated by transcription factor binding that does not affect chromatin accessibility, as footprints for key transcription factors related to immune responses and inflammation were found to be enriched in genes that showed a response to the microbiome. Other mechanisms may also explain the observed changes in gene expression. For example, others have observed that RNA processing in the host changes in response to exposure to bacteria (35, 57–59). Future studies could analyze whether the gut microbiota may modulate RNA processing directly and thereby induce a transcriptional response. In this study, we identified specific transcription factors that may play a key role in mediating the gene regulatory response to the microbiome. The recently developed CUT&RUN technique (60) could be used in the future to specifically investigate whether the microbiota induces binding of specific TFs through the use of the footprinting data we have generated to identify potential candidates. It is unlikely that changes in methylation could happen in the short time frame analyzed here and could induce the observed gene expression changes. However, a longer time course would allow investigation of methylation changes as potential mechanisms for long-term gene regulatory changes induced by the microbiome. It has been shown that epigenetic changes and changes in TF binding can be induced by the gut microbiome (31, 32), and our results confirm that they may also be among the mechanisms in human colonic epithelial cells by which the microbiota drives host gene expression.

### Conclusion.

Our results suggest that specific microbes in the microbiome may be important in regulating host gene expression in the gut and that microbes can induce changes in the expression levels of a large number of genes. Furthermore, our data support the hypothesis that the microbiome induces changes in the levels of expression of host genes that are involved in complex traits. While the mechanism of response is as yet unclear for most genes, we identified a small subset of genes whose response to microbiome exposure may be regulated by changes in chromatin accessibility. Finally, our work suggests a molecular mechanism by which supplementation of the microbiome can influence human health through changes in host cellular response. We have shown that by manipulating microbiome composition by supplementation of a single microbe, we were able to influence the host cell regulatory response in a predictable way. This work and future research will help to determine which microbes may be most beneficial in interventional therapy to improve one’s health.

## MATERIALS AND METHODS

### Extended materials and methods.

Extended materials and methods can be found in Text S1 in the supplemental material.

### Cell culture and treatment.

Experiments were conducted using primary human colonic epithelial cells (HCoEpiC; lot 9810), which we also term “colonocytes” (ScienCell catalog no. 2950). Five fecal microbiota samples were purchased from OpenBiome as live microbial suspensions in 12.5% glycerol. On the day of the experiment, medium was removed from the colonocytes and fresh antibiotic-free medium was added, followed by inoculation with microbial extract for a final microbial ratio of 10:1 (microbe/colonocyte) in each well. Additional wells containing only colonocytes were also cultured for use as controls. Control wells also received fresh antibiotic-free media when the treatment wells were being treated. While the live microbial suspension was purchased in 12.5% glycerol, the microbiota sample in the culture plate was present only in traces (0.014%); therefore, no glycerol was added to the controls. This experimental protocol was described previously (38).

After 1, 2, or 4 h, the wells were scraped on ice and the contents were pelleted and washed with cold phosphate-buffered saline (PBS) and then resuspended in lysis buffer (Dynabeads mRNA Direct kit) and stored at –80°C until extraction of colonocyte RNA was performed. Each microbiota sample was used to treat colonocytes three times in total (once for each time point). At each time point, three wells of control (untreated) colonocytes were collected as technical replicates. To identify the genes that responded to the microbiota at each time point, the five microbiota samples were treated as biological replicates (see equation 2 in Text S1). To identify the genes that responded to a specific microbiota sample, we considered the technical replicates of each microbiome collected at 3 different time points (see equation 3 in Text S1). Further details can be found below and in the supplemental material.

### *Collinsella* spike-in experiment.

Collinsella aerofaciens was purchased from ATCC (catalog no. 25986) and grown in reinforced clostridial medium (BD Biosciences; catalog no. 218081), following the manufacturer’s protocol, under anaerobic conditions. We verified that we were utilizing Collinsella aerofaciens by extracting the DNA by the use of a PowerSoil kit as described below. We then PCR amplified the 16S region using primers specific to Collinsella aerofaciens (61) (see Fig. S3F in the supplemental material).

Cell culture conditions for this experiment were the same as those described above. On the day of treatment, solutions were made with the microbiota sample (from individual 4) at 10:1 compared to the number of colonocytes and the C. aerofaciens inoculum was spiked into the microbiota sample at the following 4 dilutions: 10%, 1%, 0.1%, and 0.01%. Additional wells containing only colonocytes and colonocytes with the microbiota sample (0% Collinsella aerofaciens) were cultured as controls on the same 12-well plate. Each treatment was performed in duplicate.

Following 2 h of culturing, the wells were scraped on ice and the contents were pelleted and washed with cold PBS, resuspended in lysis buffer (Dynabeads mRNA Direct kit), and stored at –80°C until extraction of colonocyte RNA was performed (as described below).

### RNA library preparation from colonocytes.

Polyadenylated mRNAs were isolated from thawed cell lysates by the use of a Dynabeads mRNA Direct kit (Ambion) following the manufacturer’s instructions. RNA-seq libraries were prepared using a protocol modified from the NEBNext Ultradirectional (NEB) library preparation protocol to use barcodes from Bioo Scientific added by ligation, as described previously (62). The libraries were then pooled and sequenced on two lanes of an Illumina NextSeq 500 system in the Luca/Pique-Regi laboratory using high-output kits for 75 cycles to obtain paired-end (PE) reads for an average of over 40 million total reads per sample.

### RNA sequencing and alignment.

Reads were aligned to the hg19 human reference genome using STAR (63) (https://github.com/alexdobin/STAR/releases, version STAR_2.4.0h1) and the Ensemble reference transcriptome (version 75). We further removed reads with a quality score of <10 (equating to reads mapped to multiple locations) and removed duplicate reads using samtools rmdup (http://github.com/samtools/).

### Differential gene expression analysis.

To identify differentially expressed (DE) genes, we used DESeq2 (64) (R version 3.2.1, DESeq2 version 1.8.1). Gene annotations from Ensembl version 75 were used, and transcripts with fewer than 20 reads in total were discarded. coverageBed was utilized to count reads with -s to account for strandedness and -split for BED12 input. The counts for 28,107 genes (147,555 transcripts) were then utilized in DESeq2 with several models to determine changes in gene expression under various conditions. A gene was considered DE if at least one of its transcripts was DE. In order to identify genes whose level of expression changed at each time point following coculturing, we used each microbiota treatment as a replicate. With this model, we identified 1,835 genes whose level of expression changed after 1 h (70% of the genes showed increased expression levels), 4,099 genes whose level of expression changed after 2 h (53% of the genes showed increased expression levels), and 1,197 genes whose level of expression changed after 4 h (56% of the genes showed increased expression levels) (BH FDR < 10%, |logFC| > 0.25) (Fig. S1H and I).

In order to identify genes that were differentially expressed at a given time point after coculturing with a specific microbiota sample, we used an alternative model which allows analysis of different effects of each microbiome. With this model, 1,131 genes showed changes in expression levels after 1 h with any of the 5 samples, 3,240 after 2 h, and 1,060 after 4 h (BH FDR < 10%, |logFC| > 0.25 (Fig. 1B).

We next used the likelihood ratio test that is a part of DESeq2 to compare the 2 models described above in order to identify genes whose expression changes over time are determined by the individual from which the microbiota sample was taken. In this way, we identified 409 genes at a BH FDR of <10%.

In order to identify components of the microbiota samples that affect gene expression, we used a model that included the baseline abundance of each taxon as measured in the microbiota samples prior to coculturing with the human cells (after all of the samples had been rarified to the sample with the lowest read count of 141,000). For this analysis, we considered the 62 most abundant taxa (>0.1% of the total number of reads in at least one of the five samples) and all genes expressed in the colonocytes, for a total of 9,125,927 tests. We identified 588 significant taxon-gene associations (BH FDR < 10%), comprising of 46 taxa and 121 genes (see Table S1 in the supplemental material).

Finally, we analyzed the validation experiment performed with the spike-in of Collinsella aerofaciens. In order to identify genes that were differentially expressed because of the presence of Collinsella aerofaciens, we used the model that included increasing abundances of Collinsella aerofaciens. We identified 1,570 genes that showed changes in expression levels (BH FDR = 10%) (Table S1; see also Fig. S2F) depending on the abundance of Collinsella aerofaciens.

### 16S rRNA gene sequencing and analysis of the microbiome.

Half of the volume of each culturing well and the full volume of wells with microbiota samples cultured alone were used for extraction of microbial DNA by the use of a PowerSoil kit (Mo Bio Laboratories) as directed, with a few modifications (found in the supplemental material). Microbial DNA was also extracted from the uncultured microbial samples. 16S rRNA gene amplification and sequencing were performed at the University of Minnesota Genomics Center (UMGC), as described previously by Burns et al. (21).

The trimmed 16S rRNA gene sequence pairs were quality filtered (*q* score of >20, using QIIME 1.8.0), resulting in 1.41, 1.06, and 1.53 million high-quality reads for sample replicates 1, 2, and 3, respectively (65, 66). Operational taxonomic units (OTUs) were picked using the closed reference algorithm against the Greengenes database (August 2013 release) (21, 65–67). The resulting OTU table was analyzed to determine microbial community diversity using QIIME scripts and rarefaction to 141,000 reads.

We verified that the fecal samples that we utilized were similar to other healthy samples by comparing the OTUs detected to data from the Human Microbiome Project (1, 42). 16S V4 OTU and HMP V1V3 OTU tables (https://www.hmpdacc.org/HMQCP/, final OTU table) were run through QIIME’s summarize_taxa.py algorithm and consolidated at the L3 class level.

### Determining effect of colonocytes on microbiota composition.

OTUs were collapsed to the genus level using scripts in QIIME 1.9.1. In total, 292 taxa were detected across all samples and treatments. After performing filtering on the data representing the relative abundances of each taxon, we focused on 112 taxa. We used the most recent version of QIIME throughout our analysis, and the version was updated while we were working on the project. The change in version does not affect the results (running summarize_taxa.py and alpha_diversity.py from the two versions of QIIME on the OTU table produced exactly the same output tables).

To assess how each taxon changed in response to culturing with colonocytes, we ran two linear models (including or excluding the effect of colonocytes) and compared the goodness-of-fit data using a likelihood ratio test. The model yielded 13/112 taxa that changed significantly due to treatment, with a BH FDR of <10% (Fig. S2C).

### ATAC-seq in colonocytes exposed to gut microbiota.

Colonocytes were treated as described above. We treated colonocytes in a six-well plate with one of each of the five microbiota samples; as a control, one additional well was left untreated and yet was maintained under the same culturing conditions. This setup was used in duplicate, leading to the use of a total of 12 wells for the experiment. Thus, in total, the experiment consisted of five biological replicates, performed with two technical replicates each. Following 2 h of exposure to the microbiome, cells were collected by scraping the plate on ice. The contents of each well were counted to enable the removal of 50,000 cells to be used for ATAC-seq. We followed a previously reported protocol (40) to lyse 50,000 cells and prepare ATAC-seq libraries, with the exceptions that we used an Illumina Nextera index kit (catalog no. 15055290) in the PCR enrichment step and the cells were not lysed with 0.1% IGEPAL CA-630 before addition of the transposase to begin the ATAC-seq protocol. Individual library fragment distributions were assessed on an Agilent Bioanalyzer, and pooling proportions were determined using a quantitative PCR (qPCR) library quantification kit (Kapa Biosystems). Library pools were run on an Illumina NextSeq 500 desktop sequencer in the Luca/Pique-Regi laboratory. Barcoded libraries of ATAC-seq samples were pooled and sequenced in multiple sequencing runs for 130 million 38-bp PE reads (maximum, 159 million; minimum, 105 million). One sample, derived from treatment of the colonocytes with microbiota from individual 3, was removed from later analysis due to low sequencing coverage (63 million reads).

Reads were aligned to the hg19 human reference genome using HISAT2 (68) (https://github.com/, version hisat2-2.0.4) and the Ensemble reference transcriptome (hg19) with the options HISAT2 -x <genome> -1 <fastq_R1.gz> -2 <fastq_R2.gz>, where <genome> represents the location of the genome file and <fastqs_R1.gz> and <fastqs_R2.gz> represent the fastq files.

The multiple-sequencing runs were merged for each sample using samtools (version 2.25.0). We further removed reads with a quality score of <10 (equating to reads mapped to multiple locations) and removed duplicate reads using samtools rmdup (http://github.com/samtools/).

### Transcription factor binding footprints detection with CENTIPEDE.

To detect which transcription factor motifs had footprints under each set of conditions, we adapted CENTIPEDE (54) to use the fragment length information contained in ATAC-seq in the footprint model and to jointly use in parallel the treatment conditions and control conditions in order to ensure that the same footprint shape would be used for the same motifs under both sets of conditions.

As described for CENTIPEDE, we needed to start from candidate binding sites for a given motif model. For each transcription factor, we scanned the entire human genome (hg19) for matches to its DNA recognition motif using position weight matrix (PWM) models from TRANSFAC and JASPAR as previously described (54). Then, for each candidate location *l*, we collected all the ATAC-seq fragments which were partitioned into four binding groups on the basis of the fragment length as follows: (i) 39 to 99 bp, (ii) 100 to 139 bp, (iii) 140 to 179 bp, and (iv) 180 to 250 bp. For each fragment, the two Tn*5* insertion sites were calculated as the position 4 bp after the 5′ end in the 5′ to 3′ direction. Then, for each candidate motif, a matrix (***X***) was constructed to count Tn*5* insertion events; each row represented a sequence match to a motif in the genome (a motif instance) and each column a specific cleavage site at the relative base pair and orientation with respect to the motif instance. We built a matrix [${\left\{X_{l}\right\}}_{l=1}^{4}$] for each fragment length bin, each using a window a half-size *S* value of 150 bp, resulting in (2 × *S* + *W*) × 2 columns, where *W* represents the length of the motif in base pairs.

Finally, we fitted the CENTIPEDE model in a subset of instances to determine which motifs have active binding (i.e., show footprints) with a *Z* value of >5 as described previously (69). The statistical significance was assessed by calculating a Z-score corresponding to the PWM effect in the prior probability in the CENTIPEDE’s logistic hierarchical prior. Then we used CENTIPEDE and motif instances with posterior probabilities higher than 0.99 to denote locations where the transcription factors are bound.

### Identification of differentially accessible regions following inoculation with microbiota.

To identify differentially accessible regions, we used DESeq2 (64) (R version 3.2.1, DESeq2 version 1.8.1). We separated the genome into 300-bp regions, and coverageBed was used to count reads in these regions. A total of 65,000 regions with >0.25 reads per million were then utilized in DESeq2 using the following model:

(1) $$\begin{array}{ll} & \text{Gene expression}∼\text{treatment}\\ & Y_{\mathrm{jn}}=\sum_{t}{\text{β}}_{\mathrm{jt}}^{M}M_{\mathrm{tn}} \end{array}$$

where *Y_jn_* represents the internal DEseq mean accessibility parameter for region *j* and experiment *n*, *M_n_* is the treatment indicator (control or microbiome), and the ${\text{β}}_{\mathrm{jt}}^{M}$ parameter is the microbiome effect. With this model, we identified 234 regions that change in the host following exposure to the gut microbiota, with BH FDR of <20%. These regions were then compared to gene annotations from Ensembl version 75 to identify those that were within 50 kb of a transcription start site. We found enrichment for differentially accessible regions close to DE genes at 4 h (*P* value = 3.96 × 10^−4^, OR = 2.13) (Fig. 4A). In order to identify transcription factors which are important for response to microbiome exposure, we calculated enrichment scores for each active motif in regions of differentially accessible chromatin. A total of 40 motifs were found to have been significantly enriched or depleted (nominal *P* value < 0.05; see Table S4). Because we identified factors which were enriched or depleted for differentially accessible regions, we calculated a stratified FDR for regions tested for differential levels of chromatin accessibility by motif (70). For each transcription factor motif, we used the footprinting results from CENTIPEDE to classify subsets of regions containing that footprint from the DESeq2 test for differential chromatin accessibility. We then adjusted the FDR using the Benjamini-Hochberg procedure (71) for each motif separately. Combining the results from all motifs, we identified 209 regions displaying levels of differential chromatin accessibility with an FDR of <10%.

To identify transcription factor footprints enriched in differentially expressed genes, we used a logistic model for each of the 397 active factors within 50 kb upstream and downstream of each gene transcription start site as follows:

(2) $$\begin{array}{l} \log(\text{Prob. gene DE}/\text{Prob. gene not DE})={\text{β}}_{0}+{\text{β}}_{1}(\text{footprint count near gene}) \end{array}$$

We performed Benjamini-Hochberg multiple-test correction and defined significant factors as corresponding to FDR values of <10%.
