# Supplementary material for: Gut Microbiota Has a Widespread and Modifiable Effect on Host Gene Regulation
Source: mSystems. 2019 Sep 3;4(5):e00323-18. doi: 10.1128/mSystems.00323-18 (PMC6722422; doi:10.1128/mSystems.00323-18)
Supplement: TEXT S1 [file mSystems.00323-18-s0001.pdf]

Supplementary Material

— — —

Gut microbiota has a widespread and modifiable effect on host gene regulation

Allison L Richards [1], Amanda L Muehlbauer [3,4], Adnan Alazizi [1], Michael B Burns [3,4], Anthony Findley [1], Francesco Messinai [1], Trevor J Gould [3,4], Camilla Cascardo [1], Roger Pique-Regi [1,2,5], Ran Blekhman [3,4,5], Francesca Luca [1,2,5]

[1]Center for Molecular Medicine and Genetics, and

[2]Department of Obstetrics and Gynecology, Wayne State University, Detroit, Michigan 48201, USA

[3]Department of Genetics, Cell Biology and Development, and

[4]Department of Ecology, Evolution and Behavior, University of Minnesota, Minneapolis, Minnesota, USA

[5]To whom correspondence should be addressed.

Email: fluca@wayne.edu, blekhman@umn.edu, rpique@wayne.edu

Extended Materials and Methods

Cell culture and treatment

Experiments were conducted using primary human colonic epithelial cells (HCoEpiC, lot: 9810) which we also term, colonocytes (ScienCell 2950). Briefly, cells were cultured on plates or flasks coated with poly-L-lysine (PLL) (ScienCell 0413) in colonic epithelial cell medium supplemented with colonic epithelial cell growth supplement and penicillin/streptomycin, according to manufacturer's protocol (ScienCell 2951) at 37°C with 5% CO2. 24 hours before treatment, cells were changed to antibiotic-free media and moved to an incubator at 37°C, 5% CO_2, and 5% O_2 to mimic the low-oxygen environment that is present in the gut.

Fecal microbiota was purchased from OpenBiome as live microbial suspension in 12.5% glycerol. Once arrived in our lab, the extract was not thawed until the day of treatment. Fecal microbiota was collected from healthy individuals (Ind. 1: 02-028-C, Ind. 2: 0065-0016-D, Ind. 3: 0110–0006-01, Ind. 4: 0111-0014-01, Ind. 5: 0112-0002-02). Prior to treatment, the fecal microbiota was thawed at 30°C and the microbial density was assessed by spectrophotometer (OD600) (Bio-Rad SmartSpec 3000). Media was removed from the colonocytes and fresh antibiotic-free media was added, followed by inoculation with microbial extract for a final microbial ratio of 10:1 microbe:colonocyte in each well. This ratio of microbe:colonocyte was utilized based off of our previous results suggesting that this ratio is best to reconstitute a symbiotic relationship [1]. Additional wells containing only colonocytes were also cultured to be used as controls. This experimental protocol was already described in [1].

Following 1, 2 or 4 hours, the wells were scraped on ice, pelleted and washed with cold PBS and then resuspended in lysis buffer (Dynabeads mRNA Direct Kit) and stored at -80°C until extraction of colonocyte RNA.

Collinsella Spike-in Experiment

Collinsella aerofaciens was purchased from ATCC (cat#: 25986) and grown in Reinforced Clostridial Medium (BD Biosciences, cat#: 218081) following manufacturer's protocol, in anaerobic conditions. The culture was then centrifuged at 6,000 xg for 15 minutes, the media was removed and the pellet was resuspended in a 12.5% glycerol solution (in normal saline buffer). We verified that we were utilizing *Collinsella aerofaciens* by extracting the DNA with the PowerSoil kit as described below. We then PCR amplified the 16S region using primer specific to *Collinsella aerofaciens* with annealing temperature of 63°C, foward: 5'-CTTTCAGCAGGGAAGAGTCAA-3', reverse: 5'-AGCCATGCACCACCTGTATGG-3' [2]. The PCR product was run on agarose gel with a 100bp ladder (NEB, cat#: N3231S). We observed the expected band of 590bp (Figure S3F) in the *Collinsella aerofaciens* sample and we confirmed the specificity of the PCR product by assaying also a DNA sample extracted from *Odoribacter splanchnicus* (ATCC cat# 29572). We also verified that both samples contained DNA from the 16S region by performing an additional PCR on both samples with the prokaryotic 16S rDNA universal primers at an annealing temperature of 55°C, forward (27F): 5'-AGAGTTTGATCCTGGCTCAG-3', reverse (1492R): 5'-CGGTTACCTTGTTACGACTT-3' [2].

Cell culturing conditions for this experiment were the same as described above. On the day of treatment, the microbiota sample derived from individual 4 (chosen because this individual did not have a detectable amount of *Collinsella* at baseline or at any treatment time point) and the *Collinsella aerofaciens* was assessed by spectrophotometer (OD600) (Bio-Rad Smartspec 3000). Using these values, solutions were made with the microbiota sample at 10:1 to the number of colonocytes and the *Collinsella aerofaciens* was spiked into the microbiota sample at 4 dilutions: 10%, 1%, 0.1% and 0.01%. Additional wells containing only colonocytes and colonocytes with the microbiota sample (0% *Collinsella aerofaciens*) were cultured as controls on the same 12-well plate. Each treatment was performed in duplicate.

Following 2 hours of culturing, the wells were scraped on ice, pelleted and washed with cold PBS, resuspended in lysis buffer (Dynabeads mRNA Direct Kit) and stored at -80°C until extraction of colonocyte

RNA (as described above).

RNA-library preparation from colonocytes

Poly-adenylated mRNAs were isolated from thawed cell lysates using the Dynabeads mRNA Direct Kit (Ambion) and following the manufacturer's instructions. RNA-seq libraries were prepared using a protocol modified from the NEBNext Ultradirectional (NEB) library preparation protocol to use Barcodes from BIOOScientific added by ligation, as described in [3]. The individual libraries were quantified using the KAPA real-time PCR system, following the manufacturer's instructions and using a custom-made series of standards obtained from serial dilutions of the phi-X DNA (Illumina). The libraries were then pooled and sequenced on two lanes of the Illumina Next-seq 500 in the Luca/Pique laboratory using the high output kits for 75 cycles to obtain paired-end reads for an average of over 40 million total reads per sample.

RNA sequencing and Alignment

Reads were aligned to the hg19 human reference genome using STAR [4] (https://github.com/alexdobin/STAR/releases, version STAR_2.4.0h1), and the Ensemble reference transcriptome (version 75) with the following options:

```
STAR --runThreadN 12 --genomeDir <genome>
    --readFilesIn <fastqs.gz> --readFilesCommand zcat
    --outFileNamePrefix <stem> --outSAMtype BAM Unsorted
    --genomeLoad LoadAndKeep
```
(1)

where <genome> represents the location of the genome and index files, <fastqs.gz> represents that sample's fastq files, and <stem> represents the filename stem of that sample. We further removed reads with a quality score of < 10 (equating to reads mapped to multiple locations) and removed duplicate reads using samtools rmdup (http://github.com/samtools/).

Differential Gene Expression Analysis

To identify differentially expressed (DE) genes, we used `DESeq2` [5] (`R` version `3.2.1`, `DESeq2` version `1.8.1`). Gene annotations from Ensembl version 75 were used and transcripts with fewer than 20 reads total were discarded. `coverageBed` was utilized to count reads with `-s` to account for strandedness and `-split` for BED12 input. The counts were then utilized in DESeq2 with several models to determine changes in gene expression under various conditions. A gene was considered DE if at least one of its transcripts was DE. In order to identify genes that changed at each time point following co-culturing, we used each microbiota treatment as a replicate with the following model:

$$\text{Gene expression} \sim \text{time point} + \text{treatment}$$
$$Y_{jn} = \sum_t \tau_{jt} T_{tn} + \beta^M_{jt} M_{tn} \tag{2}$$

where Y_{jn} represents the internal DEseq mean gene-expression parameter for gene j and experiment n, $T_{tn} = 1$ is the time-point indicator $\{1, 2, 4\}$, M_n is the treatment indicator (control or microbiome), and τ_{jt} and β^M_{jt} parameters are time-point specific baseline and microbiome effect respectively. With this model, we identified 1,835 genes that change after 1 hour (70% of genes increase in expression), 4,099 genes after 2 hours (53% of genes increase in expression) and 1,197 genes after 4 hours (56% increase) with BH FDR $< 10\%$, $|\text{logFC}| > 0.25$ (Figure S1H and I).

In order to identify genes that were differentially expressed at a given time point after co-culturing with a specific microbiota sample, we used the following model:

$$\text{Gene expression} \sim \text{time point} + \text{microbiota sample}$$
$$Y_{jn} = \sum_t \tau_{jt} T_{tn} + \sum_{tm} \beta^M_{jtm} M_{tmn} \tag{3}$$

Compared to the first model, here we investigate the effect of each microbiome individual $m \in \{1, 2, 3, 4, 5\}$ and time-point. Note that this model allows for a different effect to each microbiome compared to the previous model. With this model 1,131 genes changed after 1 hour with any of the 5 samples, 3,240 after 2 hours and 1,060 after 4 hours with BH FDR $< 10\%$, $|\text{logFC}| > 0.25$ (Figure 1B).

We next used the likelihood ratio test that is a part of DESeq2 to compare the 2 models above in order to identify genes whose expression changes over time are determined by the individual from which the microbiota sample was taken. In this way, we identified 409 genes at BH FDR $< 10\%$.

We considered a model utilizing baseline alpha diversity to determine if diversity itself, and not necessarily any component of the microbiota, influenced host cell gene expression:

$$\begin{aligned} &\text{Gene expression} \sim \text{time point} + \text{treatment}+ \\ &\qquad\qquad\text{baseline alpha diversity} \\ &Y_{jn} = \sum_t \tau_{jt} T_{tn} + \beta_j^M M_n + \sum_m \alpha_j A_m M_{mn} \end{aligned} \tag{4}$$

Compared to the first model, we investigate the role of alpha diversity in host gene expression where α_j is the alpha diversity-specific parameter and A_m represents the alpha diversity for each microbiota m sample at baseline. Baseline alpha diversity was measured with three metrics using QIIME: Chao1, Simpson index, and Shannon index. We identified 14, 53, and 7 genes that were associated with Chao1, Simpson index, and Shannon index, respectively. However, there were no genes that were differentially expressed in all three.

In order to identify components of the microbiota samples that affect gene expression we used the following model for each gene j and taxon g:

$$\begin{aligned} &\text{Gene expression} \sim \text{time point} + \text{treatment}+ \\ &\qquad\qquad\text{baseline abundance} \\ &Y_{jn} = \sum_t \tau_{jt} T_{tn} + \beta_j^M M_n + \sum_m \gamma_{jg} G_{gm} M_{mn} \end{aligned} \tag{5}$$

Compared to the first model, we investigate the role of the baseline abundance of a given taxon in host gene expression where γ_{jg} is the taxon-specific parameter and G_{gm} represents the baseline abundance each microbiota sample m and each taxon g at baseline. Baseline abundance is the number of reads (after all samples have been rarified to the sample with the lowest read count of 141,000) for a given taxon in each of the uncultured samples. Each of the time points had the same baseline abundance. This model was

run for 62 taxa that had at least 141 reads (0.1% of the total reads in a sample) in at least one of the five uncultured samples. Comparing each taxon to all genes expressed in the colonocytes, we had 9,125,927 tests. We identified 588 significant comparisons (BH FDR $< 10\%$) comprising of 46 taxa and 121 genes (Table S1).

Finally, we analyzed the validation experiment with the spike-in of *Collinsella aerofaciens*. In order to identify genes that were differentially expressed because of the *Collinsella aerofaciens*, we used the following model:

$$\begin{aligned}\text{Gene expression} \sim\ & \text{treatment with microbiome}+ \\ & \text{relative abundance of } \textit{Collinsella aerofaciens} \\ Y_{jn} = \mu_j + \beta_j^M M_n + \gamma_j^C G_n^C M_n\end{aligned} \tag{6}$$

We investigate the role of the changing *Collinsella aerofaciens* concentration within the microbiota sample in host gene expression where μ_j is the baseline mean, β_j^M is the overall microbiome effect, and γ_j^C is the *Collinsella*-specific parameter and G_n^C represents the spiked-in abundance *Collinsella*. Where treatment with microbiome is a factor with values yes and no denoting treatment with the overall microbiome sample and relative abundance of *Collinsella aerofaciens* is a number with the values 0, 0.01, 0.1, 1 and 10 depending on the spike-in amount of each sample. We identified 1,570 genes that change expression (BH FDR = 10%, Table S1, Figure S2F) depending on the abundance of *Collinsella aerofaciens*. Note that this analysis has more power than the one across the five microbiomes.

Gene ontology analysis

We utilized GeneTrail [6] to find enrichment of gene ontology terms. We compiled a list of unique genes that changed gene expression at any of the three time points (1hr, 2hr and 4hr) and determined which GO categories were under/over-represented as compared to a list of all genes expressed in colonocytes (28,107 genes). We considered a category over/under-represented if the Benjamini-Hochberg adjusted p-value < 0.05 (Figure S1J).

Enrichment of DE genes among genome-wide association studies

We downloaded the GWAS catalog [7] (version 1.0.1) on January 5th, 2016. To identify the overlap between DE genes in our dataset and those associated with a GWAS trait, we intersected genes that contain transcripts that change significantly under a given condition with the reported genes from the GWAS catalog. We used a Fisher's exact test on a 2x2 contingency table using 2 groups: genes that contain transcripts that are DE ("DEG") and other genes that are expressed in each sample ("NOT"). We then split these groups into 2: genes that are associated with any GWAS trait ("TRAIT") and genes that are not associated with any trait in the GWAS catalog ("NOT TRAIT").

When considering specific trait enrichment among the 1,570 genes differentially expressed in the spike-in validation experiment, 35 traits were tested. These included traits that had at least 5 differentially expressed genes and 1 non-differentially expressed gene associated with it.

Heatmap and Network of Gene Expression and Microbial Abundance

Figure 2A was made by including all microbial taxa and host transcripts that were among the 588 transcript-by-taxon pairs with significant association. These were then filtered to only include transcripts that were significantly differentially expressed in at least 1 sample when each treatment is treated separately, leaving 393 transcript-by-taxon pairs including 219 transcripts (70 genes) and 28 taxa. The Spearman's ρ was then calculated for all pairs of transcripts and taxa in the heatmap (including those that did not have significant association from the taxon-based model of DESeq2).

Figure 2D was made using the Spearman's ρ of the same 393 transcript-by-taxon pairs. Transcripts with the lowest p-value (from correlation assessed by Spearman's ρ) were used to represent genes when multiple transcripts from the same gene had a significant association with a given microbial taxon (BH FDR = 10% from taxon-based model).

16S rRNA gene sequencing and analysis of the microbiome

Half of each culturing well and the full volume of wells with microbiota samples cultured alone were used for extraction of microbial DNA using the PowerSoil kit from MO BIO Laboratories as directed, with a few modifications. Microbial DNA was also extracted from the uncultured microbial samples. Briefly, the sample was spun to collect live microbes. The pellet was then resuspended in 200μL of phenol:chloroform and added to the 750μL bead solution from the PowerSoil kit. The kit protocol was then followed and the column was eluted in 60μL.

16S rRNA gene amplification and sequencing was performed at the University of Minnesota Genomics Center (UMGC), as described in Burns et al. [8]. Briefly, DNA isolated from the fecal microbiota was quantified by qPCR, and the V5-V6 regions of the 16S rRNA gene were PCR amplified with forward primer 5'-TCGTCGGCAGCGTCAGATGTGTATAAGAGACAG**RGGATTAGATACCC**-3', and reverse primer 5'-GTCTCGTGGGCTCGGAGATGTGTATAAGAGACAG**CGACRRCCATGCANCACCT**-3', where the portion in bold is 16S-specific. Nextera indexing primers were added in the next PCR using the V5F primer 5'-AATGATACGGCGACCACCGAGATCTACAC[i5]TCGTCGGCAGCGTC-3', and V6R 5'-CAAGCAGAAGACGGCATACGAGAT[i7]GTCTCGTGGGCTCGG-3', where [i5] and [i7] refer to the index sequences used by Illumina. This PCR was carried out using the KAPA HiFidelity Hot Start polymerase (Kapa Biosystems) for 20 cycles. The amplicons were then diluted 1:100 and used as input for a second PCR using different combinations of forward and reverse indexing primers for another 10 cycles. The pooled, size-selected product was diluted to 8pM, spiked with 15% PhiX and loaded onto an Illumina MiSeq instrument to generate the 16S rRNA gene sequences. Samples were heat denatured at 96 °C for 2 minutes prior to loading, and a MiSeq 600 cycle v3 kit was used to sequence the sample, resulting in 2.2 million raw reads per sample, on average. Barcodes were removed from the sample reads by UMGC and the Nextera adaptors were trimmed using CutAdapt 1.8.1.

The trimmed 16S rRNA gene sequence pairs were quality filtered (q-score $>$ 20, using QIIME 1.8.0) resulting in 1.41, 1.06, and 1.53 million high quality reads for sample replicates 1, 2, and 3, respectively [9,

10]. OTUs were picked using the closed reference algorithm against the Greengenes database (August, 2013 release) [8, 9, 10, 11]. The resulting OTU table was analyzed to determine microbial community diversity using QIIME scripts and rarefying to 141,000 reads. Rarefaction plot was generated using QIIME on the five uncultured samples (Figure S1B).

We verified that the fecal samples we utilized were similar to other healthy samples by comparing the OTUs detected to the Human Microbiome Project data [12, 13]. 16S V4 OTU and HMP V1V3 OTU tables (`https://www.hmpdacc.org/HMQCP/`, final OTU table) were run through QIIME's `summarize_taxa.py` and consolidated at the L3 class level. Tables were merged by most prevalent classes (13) with the remaining taxa merged into other representing $< 1\%$ of the total count on average. A Bray Curtis distance matrix and PCoA were performed in the R vegan package and plots were created with ggplot2, grid and gridextra libraries (Figure S1E). HMP fecal samples colored red, study samples colored yellow and all other HMP samples colored grey.

Determining Effect of Colonocytes on Microbiota Composition

OTUs were collapsed to the genera level using scripts in QIIME 1.9.1. In total, 292 taxa were detected across all samples and treatments. To filter the number of taxa, the relative abundances were summed for each taxon, and taxa with summed relative abundance greater than 0.003 were kept in the linear model. This yields 85 taxa of interest. To account for taxa that may be abundant at only at a particular time point, relative abundances of taxa were summed at each time point and kept if they had a total relative abundance greater than 0.0005. For time point 0, this threshold was 0.00025. The additional step yields 27 taxa; these were added onto the list of 85 for a total of 112 taxa.

To identify taxa that change significantly over time we used a linear model. Relative abundances of the 112 taxa of interest were normalized using an arcsine-square root transformation. In the linear model, "treatment" is binary and refers to microbiome samples grown with colonocytes or without colonocytes. In the model, this is treated as a factor. "Individual" refers to the individual microbiome being studied

and is treated as a factor. Time is treated as a factor variable, with possible values of 1, 2 and 4 hours, in order to allow for changes that are not necessarily linear over time.

To assess how each taxon changed in response to culturing with colonocytes, we ran two linear models and compared the goodness of fit using a likelihood ratio test. P-values from the likelihood ratio test were extracted and adjusted using the Benjamini-Hochberg procedure.

Null Model

$$\text{Transformed Relative Abundance of Taxa} \sim \text{Individual} + \text{Time}$$
$$G_{gn} = \sum_t \tau_{gt} T_{tn} + \sum_m \beta^M_{gm} M_{mn} \tag{7}$$

Alternative Model

$$\text{Transformed Relative Abundance of Taxa} \sim \text{Individual}+$$
$$\text{Treatment} + \text{Time}$$
$$G_{gn} = \sum_t \tau_{gt} T_{tn} + \sum_m \beta^M_{gm} M_{mn} + \gamma_g K_n \tag{8}$$

where G_{gn} represents the transformed relative abundance of taxa parameter for taxon g and experiment n, $T_{tn} = 1$ is the time-point indicator $\{1, 2, 4\}$, M_{mn} is the treatment indicator $m \in \{1, 2, 3, 4, 5\}$, and τ_{gt} and β^M_{gm} parameters are time-point specific baseline and individual microbiome effect respectively. The alternative model includes K_n as an indicator of the presence of colonocytes, γ_g is the colonocyte effect on each taxon. The model yielded 13/112 taxa that change significantly due to treatment with a BH FDR $< 10\%$ (Figure S2C).

Accession numbers for sequencing data

16S rRNA sequencing data and RNA sequencing data of colonocytes in all conditions have been deposited to the SRA (SRP150967).

Additional Results and Analyses

Characteristics of the Gut Microbiome

Thirty-five microbial samples were assessed with 16S rRNA gene sequencing, including the five uncultured microbiota samples, each of these five cultured alone (without colonocytes) for 1, 2, and 4 hours, and co-cultured with colonocytes for 1, 2 and 4 hours. In total, there were 7 samples from each original microbiome, and 35 samples overall. QIIME 1.8.0 was used to analyze the resulting sequences [9]. We found that the relative abundances of each taxa were most strongly affected by the individual from which the sample was derived and did not change dramatically over time (Figure S1A and C). We also found that the samples clustered by individual using weighted UniFrac, although more weakly (Figure S1D). The microbiome maintains its composition during culture and our sequencing depth is able to capture this variation (Figure S1B). These results demonstrate that the gut microbiota samples are typical and unaffected by culturing for up to 4 hours.

Response of Human Colonocytes to Inoculation with Microbiome

When we consider how the microbiota influences the host cell response, we identify 3,904 genes that are differentially expressed in at least one sample at 1, 2 or 4 hours compared to control colonocytes. In order to gain power to identify shared differentially expressed genes, we next utilized a model where treatments at a given time point are considered replicates and compared to control colonocyte samples. In this model, we identified 4,099 genes (BH FDR $< 10\%$, $|\log_2 \mathrm{FC}| > 0.25$) that change expression consistently across the five microbiota treatments at 2 hours, while over 1,000 change consistently at 1 and 4 hours (examples in Figure 1D, Figure S1H and I, Table S1). We were unable to identify any genes that were differentially expressed based on diversity of the microbiota sample at baseline, independent of diversity measure. However, we identified 121 genes that were associated with microbial taxa at baseline.

Effect of Human Colonocytes on Microbiome Composition

We assessed the microbial composition of the individual microbiome samples grown with and without colonocytes. Microbiome samples grown with colonocytes were collected concurrently and from the same wells that RNA-seq data was generated. We identified 13 taxa that showed varying abundance dependent on the presence of host cells (Figure S2A, B and C). These data demonstrate that interaction with the host affects microbial communities. We co-culture the colonocytes and microbiota for up to four hours, but it is likely that in vivo, the various species will eventually come to an equilibrium which can then be maintained in the host organism and will reflect both host genetic variation and diet. When this equilibrium is disrupted, previous studies have shown that disease and infection may occur [14, 15, 16, 17, 18].

References

[1] Richards, A.L., Burns, M.B., Alazizi, A., Barreiro, L.B., Pique-Regi, R., Blekhman, R., Luca, F.: Genetic and transcriptional analysis of human host response to healthy gut microbiota. mSystems **1**(4) (2016)

[2] Kageyama, A., Sakamoto, M., Benno, Y.: Rapid identification and quantification of Collinsella aerofaciens using PCR. FEMS Microbiol. Lett. **183**(1), 43–47 (2000)

[3] Moyerbrailean, G.A., Davis, G.O., Harvey, C.T., Watza, D., Wen, X., Pique-Regi, R., Luca, F.: A high-throughput RNA-seq approach to profile transcriptional responses. Sci Rep **5**, 14976 (2015)

[4] Dobin, A., Davis, C.A., Schlesinger, F., Drenkow, J., Zaleski, C., Jha, S., Batut, P., Chaisson, M., Gingeras, T.R.: STAR: ultrafast universal RNA-seq aligner. Bioinformatics **29**(1), 15–21 (2013)

[5] Love, M.I., Huber, W., Anders, S.: Moderated estimation of fold change and dispersion for RNA-seq data with DESeq2. Genome Biol. **15**(12), 550 (2014)

[6] Backes, C., Keller, A., Kuentzer, J., Kneissl, B., Comtesse, N., Elnakady, Y.A., Muller, R., Meese, E., Lenhof, H.P.: GeneTrail–advanced gene set enrichment analysis. Nucleic Acids Res. **35**(Web Server issue), 186–192 (2007)

[7] Welter, D., MacArthur, J., Morales, J., Burdett, T., Hall, P., Junkins, H., Klemm, A., Flicek, P., Manolio, T., Hindorff, L., Parkinson, H.: The NHGRI GWAS Catalog, a curated resource of SNP-trait associations. Nucleic Acids Res. **42**(Database issue), 1001–1006 (2014)

[8] Burns, M.B., Lynch, J., Starr, T.K., Knights, D., Blekhman, R.: Virulence genes are a signature of the microbiome in the colorectal tumor microenvironment. Genome Med **7**(1), 55 (2015)

[9] Caporaso, J.G., Kuczynski, J., Stombaugh, J., Bittinger, K., Bushman, F.D., Costello, E.K., Fierer, N., Pena, A.G., Goodrich, J.K., Gordon, J.I., Huttley, G.A., Kelley, S.T., Knights, D., Koenig, J.E.,

Ley, R.E., Lozupone, C.A., McDonald, D., Muegge, B.D., Pirrung, M., Reeder, J., Sevinsky, J.R., Turnbaugh, P.J., Walters, W.A., Widmann, J., Yatsunenko, T., Zaneveld, J., Knight, R.: QIIME allows analysis of high-throughput community sequencing data. Nat. Methods **7**(5), 335–336 (2010)

[10] Navas-Molina, J.A., Peralta-Sanchez, J.M., Gonzalez, A., McMurdie, P.J., Vazquez-Baeza, Y., Xu, Z., Ursell, L.K., Lauber, C., Zhou, H., Song, S.J., Huntley, J., Ackermann, G.L., Berg-Lyons, D., Holmes, S., Caporaso, J.G., Knight, R.: Advancing our understanding of the human microbiome using QIIME. Meth. Enzymol. **531**, 371–444 (2013)

[11] DeSantis, T.Z., Hugenholtz, P., Larsen, N., Rojas, M., Brodie, E.L., Keller, K., Huber, T., Dalevi, D., Hu, P., Andersen, G.L.: Greengenes, a chimera-checked 16S rRNA gene database and workbench compatible with ARB. Appl. Environ. Microbiol. **72**(7), 5069–5072 (2006)

[12] Methe, B.A., Nelson, K.E., Pop, M., Creasy, H.H., Giglio, M.G., Huttenhower, C., Gevers, D., Petrosino, J.F., Abubucker, S., Badger, J.H., Chinwalla, A.T., Earl, A.M., FitzGerald, M.G., Fulton, R.S., Hallsworth-Pepin, K., Lobos, E.A., Madupu, R., Magrini, V., Martin, J.C., Mitreva, M., Muzny, D.M., Sodergren, E.J., Versalovic, J., Wollam, A.M., Worley, K.C., Wortman, J.R., Young, S.K., Zeng, Q., Aagaard, K.M., Abolude, O.O., Allen-Vercoe, E., Alm, E.J., Alvarado, L., Andersen, G.L., Anderson, S., Appelbaum, E., Arachchi, H.M., Armitage, G., Arze, C.A., Ayvaz, T., Baker, C.C., Begg, L., Belachew, T., Bhonagiri, V., Bihan, M., Blaser, M.J., Bloom, T., Bonazzi, V.R., Brooks, P., Buck, G.A., Buhay, C.J., Busam, D.A., Campbell, J.L., Canon, S.R., Cantarel, B.L., Chain, P.S., Chen, I.M., Chen, L., Chhibba, S., Chu, K., Ciulla, D.M., Clemente, J.C., Clifton, S.W., Conlan, S., Crabtree, J., Cutting, M.A., Davidovics, N.J., Davis, C.C., DeSantis, T.Z., Deal, C., Delehaunty, K.D., Dewhirst, F.E., Deych, E., Ding, Y., Dooling, D.J., Dugan, S.P., Dunne, W., Durkin, A., Edgar, R.C., Erlich, R.L., Farmer, C.N., Farrell, R.M., Faust, K., Feldgarden, M., Felix, V.M., Fisher, S., Fodor, A.A., Forney, L., Foster, L., Di Francesco, V., Friedman, J., Friedrich, D.C., Fronick, C.C., Fulton, L.L., Gao, H., Garcia, N., Giannoukos, G., Giblin, C., Giovanni, M.Y., Goldberg, J.M., Goll, J.,

Gonzalez, A., Griggs, A., Gujja, S., Haas, B.J., Hamilton, H.A., Harris, E.L., Hepburn, T.A., Herter, B., Hoffmann, D.E., Holder, M.E., Howarth, C., Huang, K.H., Huse, S.M., Izard, J., Jansson, J.K., Jiang, H., Jordan, C., Joshi, V., Katancik, J.A., Keitel, W.A., Kelley, S.T., Kells, C., Kinder-Haake, S., King, N.B., Knight, R., Knights, D., Kong, H.H., Koren, O., Koren, S., Kota, K.C., Kovar, C.L., Kyrpides, N.C., La Rosa, P.S., Lee, S.L., Lemon, K.P., Lennon, N., Lewis, C.M., Lewis, L., Ley, R.E., Li, K., Liolios, K., Liu, B., Liu, Y., Lo, C.C., Lozupone, C.A., Lunsford, R., Madden, T., Mahurkar, A.A., Mannon, P.J., Mardis, E.R., Markowitz, V.M., Mavrommatis, K., McCorrison, J.M., McDonald, D., McEwen, J., McGuire, A.L., McInnes, P., Mehta, T., Mihindukulasuriya, K.A., Miller, J.R., Minx, P.J., Newsham, I., Nusbaum, C., O'Laughlin, M., Orvis, J., Pagani, I., Palaniappan, K., Patel, S.M., Pearson, M., Peterson, J., Podar, M., Pohl, C., Pollard, K.S., Priest, M.E., Proctor, L.M., Qin, X., Raes, J., Ravel, J., Reid, J.G., Rho, M., Rhodes, R., Riehle, K.P., Rivera, M.C., Rodriguez-Mueller, B., Rogers, Y.H., Ross, M.C., Russ, C., Sanka, R.K., Sankar, P., Sathirapongsasuti, J., Schloss, J.A., Schloss, P.D., Schmidt, T.M., Scholz, M., Schriml, L., Schubert, A.M., Segata, N., Segre, J.A., Shannon, W.D., Sharp, R.R., Sharpton, T.J., Shenoy, N., Sheth, N.U., Simone, G.A., Singh, I., Smillie, C.S., Sobel, J.D., Sommer, D.D., Spicer, P., Sutton, G.G., Sykes, S.M., Tabbaa, D.G., Thiagarajan, M., Tomlinson, C.M., Torralba, M., Treangen, T.J., Truty, R.M., Vishnivetskaya, T.A., Walker, J., Wang, L., Wang, Z., Ward, D.V., Warren, W., Watson, M.A., Wellington, C., Wetterstrand, K.A., White, J.R., Wilczek-Boney, K., Wu, Y.Q., Wylie, K.M., Wylie, T., Yandava, C., Ye, L., Ye, Y., Yooseph, S., Youmans, B.P., Zhang, L., Zhou, Y., Zhu, Y., Zoloth, L., Zucker, J.D., Birren, B.W., Gibbs, R.A., Highlander, S.K., Weinstock, G.M., Wilson, R.K., White, O.: A framework for human microbiome research. Nature **486**(7402), 215–221 (2012)

[13] Huttenhower, C., Gevers, D., Knight, R., Abubucker, S., Badger, J.H., Chinwalla, A.T., Creasy, H.H., Earl, A.M., FitzGerald, M.G., Fulton, R.S., Giglio, M.G., Hallsworth-Pepin, K., Lobos, E.A., Madupu, R., Magrini, V., Martin, J.C., Mitreva, M., Muzny, D.M., Sodergren, E.J., Versalovic, J., Wollam, A.M., Worley, K.C., Wortman, J.R., Young, S.K., Zeng, Q., Aagaard, K.M., Abolude, O.O.,

Allen-Vercoe, E., Alm, E.J., Alvarado, L., Andersen, G.L., Anderson, S., Appelbaum, E., Arachchi, H.M., Armitage, G., Arze, C.A., Ayvaz, T., Baker, C.C., Begg, L., Belachew, T., Bhonagiri, V., Bihan, M., Blaser, M.J., Bloom, T., Bonazzi, V., Brooks, J., Buck, G.A., Buhay, C.J., Busam, D.A., Campbell, J.L., Canon, S.R., Cantarel, B.L., Chain, P.S., Chen, I.M., Chen, L., Chhibba, S., Chu, K., Ciulla, D.M., Clemente, J.C., Clifton, S.W., Conlan, S., Crabtree, J., Cutting, M.A., Davidovics, N.J., Davis, C.C., DeSantis, T.Z., Deal, C., Delehaunty, K.D., Dewhirst, F.E., Deych, E., Ding, Y., Dooling, D.J., Dugan, S.P., Dunne, W.M., Durkin, A., Edgar, R.C., Erlich, R.L., Farmer, C.N., Farrell, R.M., Faust, K., Feldgarden, M., Felix, V.M., Fisher, S., Fodor, A.A., Forney, L.J., Foster, L., Di Francesco, V., Friedman, J., Friedrich, D.C., Fronick, C.C., Fulton, L.L., Gao, H., Garcia, N., Giannoukos, G., Giblin, C., Giovanni, M.Y., Goldberg, J.M., Goll, J., Gonzalez, A., Griggs, A., Gujja, S., Haake, S.K., Haas, B.J., Hamilton, H.A., Harris, E.L., Hepburn, T.A., Herter, B., Hoffmann, D.E., Holder, M.E., Howarth, C., Huang, K.H., Huse, S.M., Izard, J., Jansson, J.K., Jiang, H., Jordan, C., Joshi, V., Katancik, J.A., Keitel, W.A., Kelley, S.T., Kells, C., King, N.B., Knights, D., Kong, H.H., Koren, O., Koren, S., Kota, K.C., Kovar, C.L., Kyrpides, N.C., La Rosa, P.S., Lee, S.L., Lemon, K.P., Lennon, N., Lewis, C.M., Lewis, L., Ley, R.E., Li, K., Liolios, K., Liu, B., Liu, Y., Lo, C.C., Lozupone, C.A., Lunsford, R., Madden, T., Mahurkar, A.A., Mannon, P.J., Mardis, E.R., Markowitz, V.M., Mavromatis, K., McCorrison, J.M., McDonald, D., McEwen, J., McGuire, A.L., McInnes, P., Mehta, T., Mihindukulasuriya, K.A., Miller, J.R., Minx, P.J., Newsham, I., Nusbaum, C., O'Laughlin, M., Orvis, J., Pagani, I., Palaniappan, K., Patel, S.M., Pearson, M., Peterson, J., Podar, M., Pohl, C., Pollard, K.S., Pop, M., Priest, M.E., Proctor, L.M., Qin, X., Raes, J., Ravel, J., Reid, J.G., Rho, M., Rhodes, R., Riehle, K.P., Rivera, M.C., Rodriguez-Mueller, B., Rogers, Y.H., Ross, M.C., Russ, C., Sanka, R.K., Sankar, P., Sathirapongsasuti, J., Schloss, J.A., Schloss, P.D., Schmidt, T.M., Scholz, M., Schriml, L., Schubert, A.M., Segata, N., Segre, J.A., Shannon, W.D., Sharp, R.R., Sharpton, T.J., Shenoy, N., Sheth, N.U., Simone, G.A., Singh, I., Smillie, C.S., Sobel, J.D., Sommer, D.D., Spicer, P., Sutton, G.G., Sykes, S.M., Tabbaa, D.G., Thiagarajan, M., Tomlinson, C.M., Torralba, M., Treangen,

T.J., Truty, R.M., Vishnivetskaya, T.A., Walker, J., Wang, L., Wang, Z., Ward, D.V., Warren, W., Watson, M.A., Wellington, C., Wetterstrand, K.A., White, J.R., Wilczek-Boney, K., Wu, Y., Wylie, K.M., Wylie, T., Yandava, C., Ye, L., Ye, Y., Yooseph, S., Youmans, B.P., Zhang, L., Zhou, Y., Zhu, Y., Zoloth, L., Zucker, J.D., Birren, B.W., Gibbs, R.A., Highlander, S.K., Methe, B.A., Nelson, K.E., Petrosino, J.F., Weinstock, G.M., Wilson, R.K., White, O.: Structure, function and diversity of the healthy human microbiome. Nature **486**(7402), 207–214 (2012)

[14] Chassaing, B., Darfeuille-Michaud, A.: The commensal microbiota and enteropathogens in the pathogenesis of inflammatory bowel diseases. Gastroenterology **140**(6), 1720–1728 (2011)

[15] Hansen, R., Russell, R.K., Reiff, C., Louis, P., McIntosh, F., Berry, S.H., Mukhopadhya, I., Bisset, W.M., Barclay, A.R., Bishop, J., Flynn, D.M., McGrogan, P., Loganathan, S., Mahdi, G., Flint, H.J., El-Omar, E.M., Hold, G.L.: Microbiota of de-novo pediatric IBD: increased Faecalibacterium prausnitzii and reduced bacterial diversity in Crohn's but not in ulcerative colitis. Am. J. Gastroenterol. **107**(12), 1913–1922 (2012)

[16] de Oliveira, G.L.V., Leite, A.Z., Higuchi, B.S., Gonzaga, M.I., Mariano, V.S.: Intestinal dysbiosis and probiotic applications in autoimmune diseases. Immunology (2017)

[17] Lin, L., Zhang, J.: Role of intestinal microbiota and metabolites on gut homeostasis and human diseases. BMC Immunol. **18**(1), 2 (2017)

[18] Turnbaugh, P.J., Backhed, F., Fulton, L., Gordon, J.I.: Diet-induced obesity is linked to marked but reversible alterations in the mouse distal gut microbiome. Cell Host Microbe **3**(4), 213–223 (2008)
