# Supplementary figures and images for: Gut Microbiota Has a Widespread and Modifiable Effect on Host Gene Regulation
Source: mSystems. 2019 Sep 3;4(5):e00323-18. doi: 10.1128/mSystems.00323-18 (PMC6722422; doi:10.1128/mSystems.00323-18)

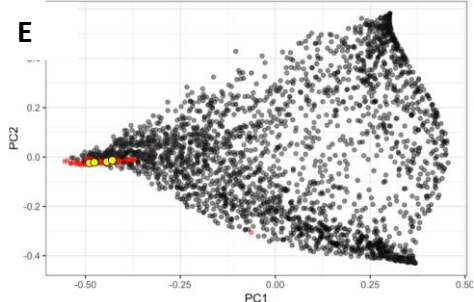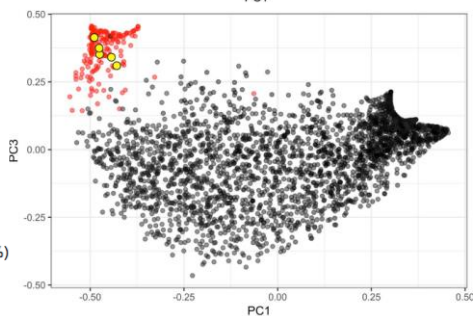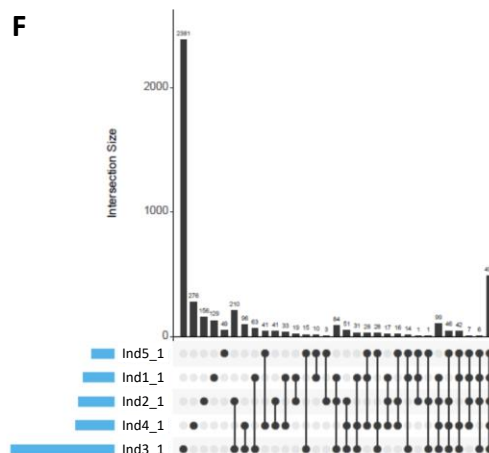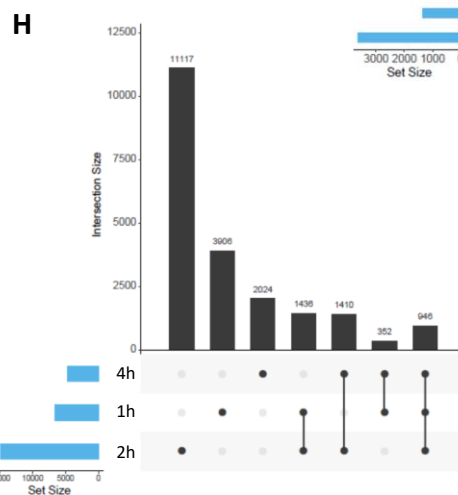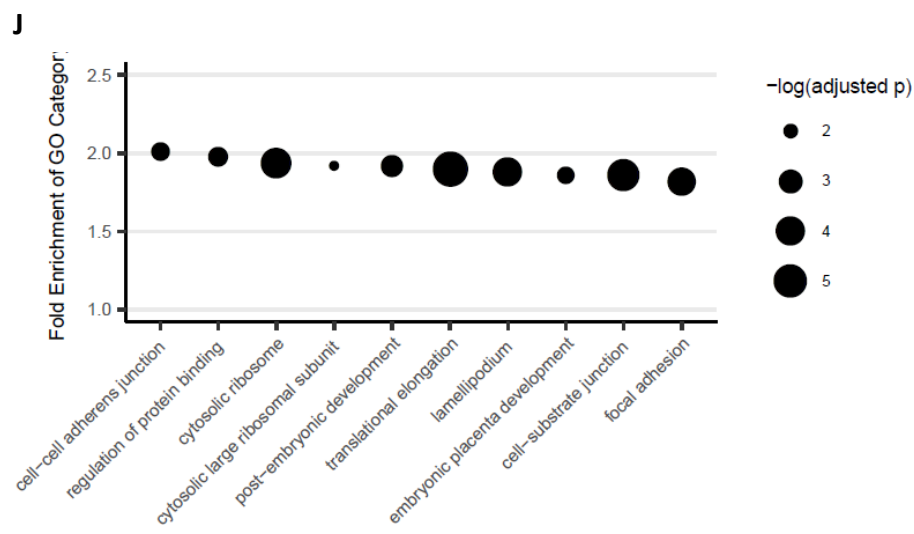

Supplement: FIG S1 [file mSystems.00323-18-sf001.pdf]

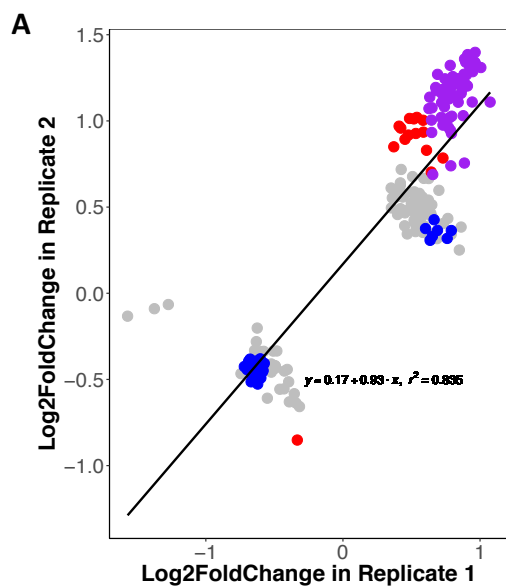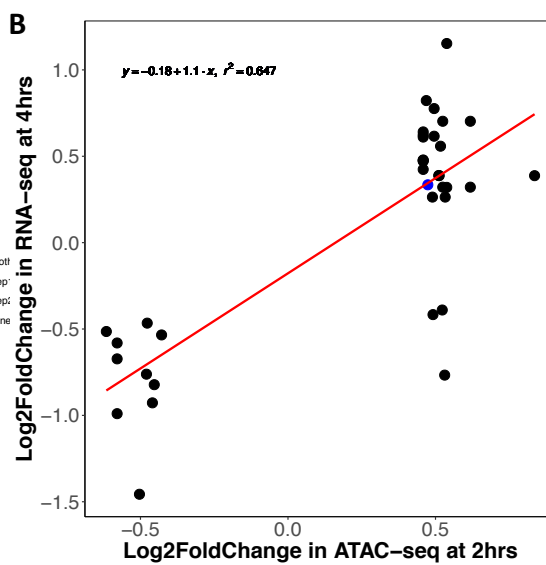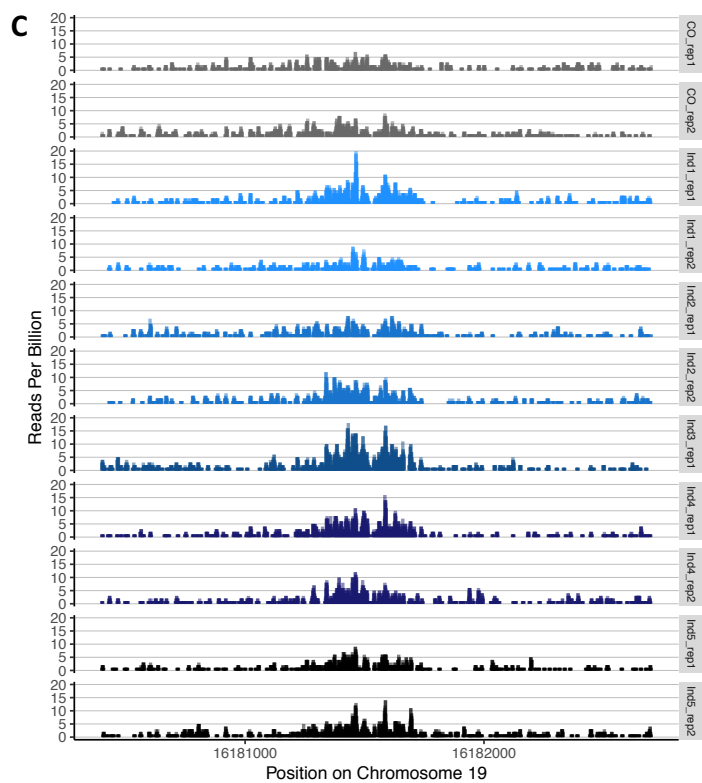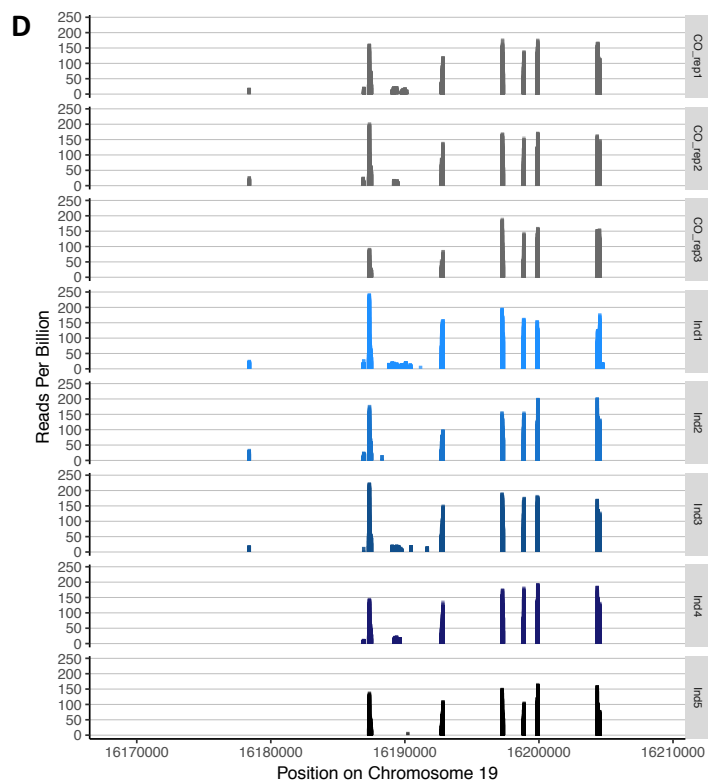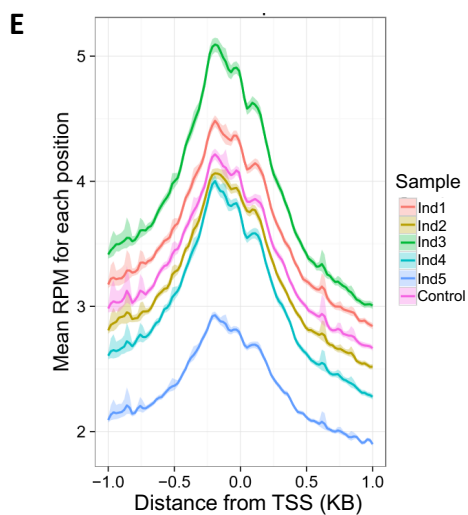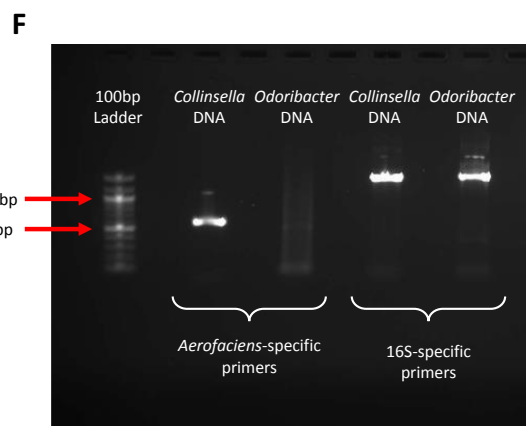

Supplement: FIG S3 [file mSystems.00323-18-sf003.pdf]
