# Supplementary material for: Gut Microbiota Has a Widespread and Modifiable Effect on Host Gene Regulation
Source: mSystems. 2019 Sep 3;4(5):e00323-18. doi: 10.1128/mSystems.00323-18 (PMC6722422; doi:10.1128/mSystems.00323-18)

A

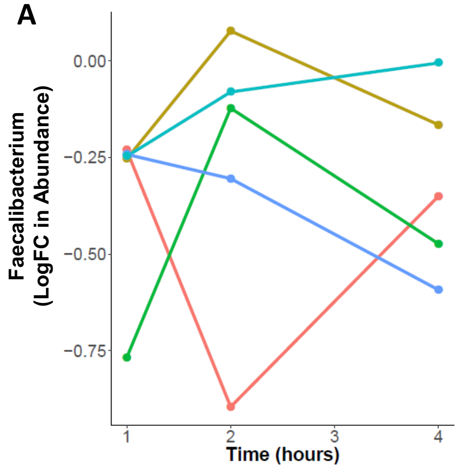

B

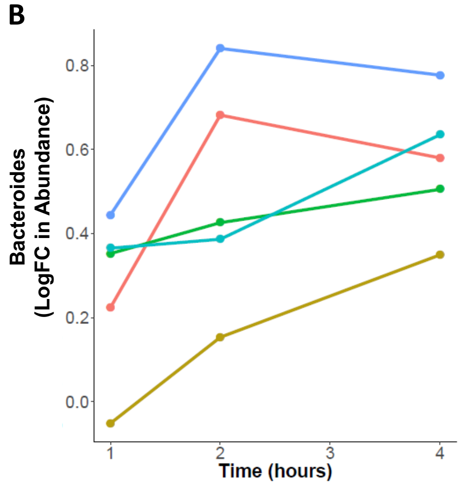

C

Bacteria Taxa	p-value	BH adjusted p-value
k__Archaea.p__Euryarchaeota.c__DSEG.o__DHVE3.f__.g__.	1.55E-09	1.73E-07
k__Bacteria.p__Bacteroidetes.c__Bacteroidia.o__Bacteroidales.f__Prevotellaceae.g__Prevotella	2.59E-07	1.45E-05
k__Bacteria.p__Bacteroidetes.c__Bacteroidia.o__Bacteroidales.f__Bacteroidaceae.g__Bacteroides	1.61E-05	0.000480663
k__Bacteria.p__Cyanobacteria.c__Chloroplast.o__Streptophyta.f__.g__	1.72E-05	0.000480663
k__Bacteria.p__Bacteroidetes.c__Bacteroidia.o__Bacteroidales.f__Porphyromonadaceae.g__Candidatus.Azobacteroides	0.000288621	0.006465103
k__Bacteria.p__Proteobacteria.c__Betaproteobacteria.o__Burkholderiales.f__Comamonadaceae.g__.	0.000960123	0.017922301
k__Bacteria.p__Firmicutes.c__Clostridia.o__Clostridiales.f__Clostridiaceae.g__	0.001734073	0.027745174
k__Bacteria.p__Actinobacteria.c__Actinobacteria.o__Bifidobacteriales.f__Bifidobacteriaceae.g__Bifidobacterium	0.003306716	0.046294026
k__Bacteria.p__Firmicutes.c__Clostridia.o__Clostridiales.f__Ruminococcaceae.g__Faecalibacterium	0.005076328	0.063172086
k__Bacteria.p__Bacteroidetes.c__Bacteroidia.o__Bacteroidales.f__Bacteroidaceae.g__5.7N15	0.006464096	0.065816246
k__Bacteria.p__Bacteroidetes.c__Cytophagia.o__Cytophagales.f__Cytophagaceae.g__	0.006431979	0.065816246
k__Bacteria.p__Proteobacteria.c__Alphaproteobacteria.o__Rhizobiales.f__Methylobacteriaceae.g__Methylobacterium	0.008861207	0.079584783
k__Bacteria.p__Verrucomicrobia.c__Verrucomicrobiae.o__Verrucomicrobiales.f__Verrucomicrobiaceae.g__Akkermansia	0.009237519	0.079584783

D

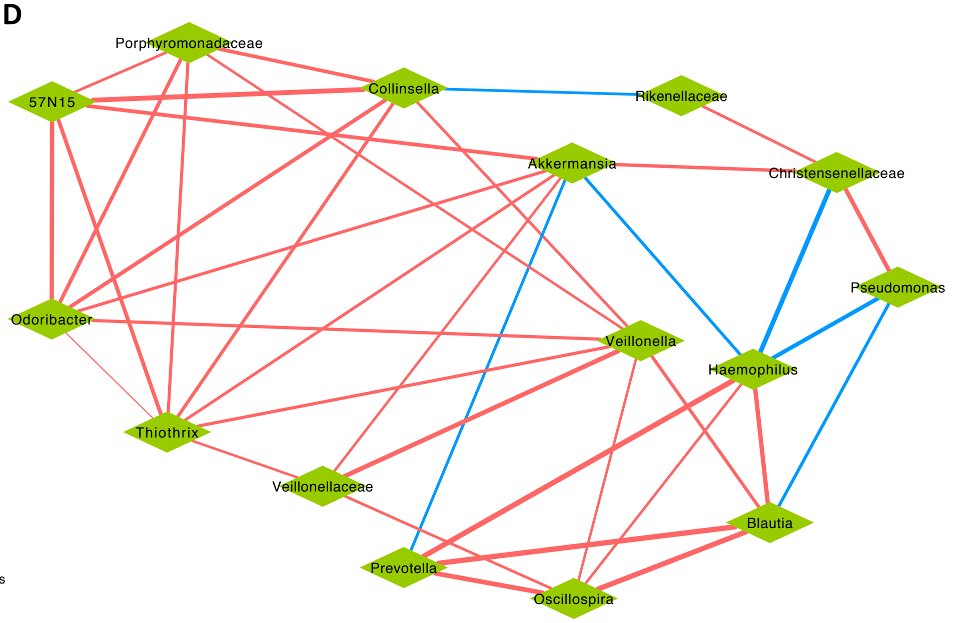

E

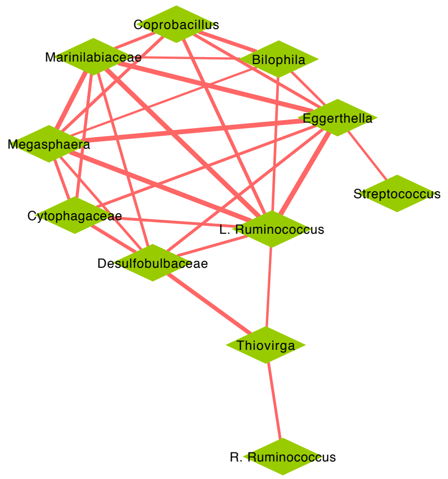

F

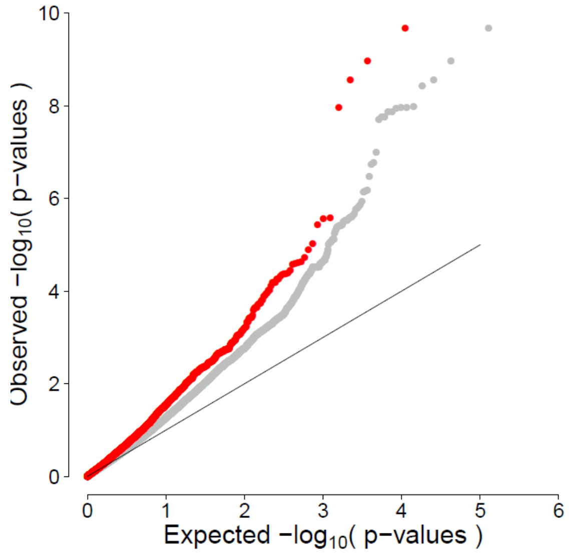

Supplement: FIG S2 [file mSystems.00323-18-sf002.pdf]
